# Coordination Polymers of Polyphenyl-Substituted Potassium Cyclopentadienides

**DOI:** 10.3390/molecules27227725

**Published:** 2022-11-09

**Authors:** Pavel D. Komarov, Kirill P. Birin, Alexander A. Vinogradov, Evgenia A. Varaksina, Lada N. Puntus, Konstantin A. Lyssenko, Andrei V. Churakov, Ilya E. Nifant’ev, Mikhail E. Minyaev, Dmitrii M. Roitershtein

**Affiliations:** 1 A.V. Topchiev Institute of Petrochemical Synthesis, Russian Academy of Sciences, 29 Leninsky Prospect, 119991 Moscow, Russia; 2 A.N. Frumkin Institute of Physical Chemistry and Electrochemistry, Russian Academy of Sciences, 31 Leninsky Prospect, Building 4, 119071 Moscow, Russia; 3 P.N. Lebedev Physical Institute, Russian Academy of Sciences, 53 Leninsky Prospect, 119991 Moscow, Russia; 4 V.A. Kotel’nikov Institute of Radioengineering and Electronics, Russian Academy of Sciences, 1 Vvedenskogo sq, 141120 Moscow, Russia; 5 G.V. Plekhanov Russian University of Economics, 36 Stremyanny Per., 117997 Moscow, Russia; 6 Chemistry Department, M.V. Lomonosov Moscow State University, 1 Leninskie Gory Str., Building 3, 119991 Moscow, Russia; 7 N.S. Kurnakov Institute of General and Inorganic Chemistry, Russian Academy of Sciences, 31 Leninsky Prospect, 119991 Moscow, Russia; 8 N.D. Zelinsky Institute of Organic Chemistry, Russian Academy of Sciences, 47 Leninsky Prospect, 119991 Moscow, Russia; 9 National Research University Higher School of Economics, 20 Miasnitskaya Street, 101000 Moscow, Russia

**Keywords:** potassium, cyclopentadienyl, diphenylcyclopentadiene, triphenylcyclopentadiene, coordination polymer, crystal structure, luminescence, charge transfer

## Abstract

A series of potassium salts of di- and tri-arylsubstituted cyclopentadienes has been obtained by the metalation of the corresponding cyclopentadienes with benzylpotassium in THF media. Crystals of all compounds, afforded by recrystallization from THF/hexane, diglyme-THF/hexane and toluene/hexane mixtures, have been studied by X-ray diffraction. All studied potassium cyclopentadienides exhibit the luminescence at room temperature and overall quantum yield of photoluminescence for potassium salt of diarylsubstituted cyclopentadiene is 18%.

## 1. Introduction

The cyclopentadienyl anion, its substituted derivatives and various analogs (e.g., indenyl, fluorenyl, etc.) are probably the most frequently used ligands in organometallic chemistry since the middle of the last century [1,2,3,4]. The synthesis of cyclopentadienyl complexes of d- and f-metals usually requires the use of cyclopentadienyl derivatives of alkali metals. Sodium and lithium cyclopentadienides are the most popular, while potassium salts of cyclopentadienes are used much less frequently in the synthesis of f-element cyclopentadienyl complexes [5]. Metalation of various cyclopentadienes, using organopotassium compounds, is more attractive due to the greater basicity of organopotassium compounds compared to RLi and RNa compounds [6]. Despite this, potassium cyclopentadienides still find limited use in synthesis, probably due to traditional ideas about the low availability of organopotassium compounds compared to lithium and sodium derivatives. Meanwhile, recently introduced convenient methods for the synthesis of different organopotassium compounds, for example, benzyl potassium, have significantly increased the availability of potassium cyclopentadienides, which makes them increasingly popular in the synthesis of Cp derivatives of d- and f-organometallic compounds [7,8,9]. However, little is known about the structure of these precursors, sometimes even the exact composition of these reagents (for example, the number of coordinated donor ligands) is questionable. The availability of information on the structure and properties of various potassium cyclopentadienides should promote the development of methods for the synthesis of these important precursors.

**Scheme 1:**
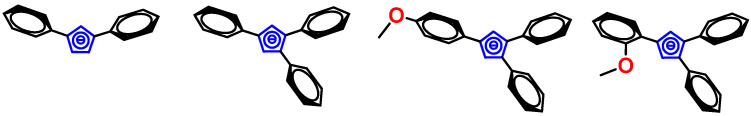
Structures of the cyclopentadienyl-anions discussed in this article.

In this work, we studied the structural features of a number of polyaryl-substituted potassium cyclopentadienides (Scheme 1), as well as their main photophysical properties, which may be useful in comparative studies of the photophysical properties of lanthanide cyclopentadienyl complexes obtained from the corresponding potassium salts discussed here.

## 2. Results and Discussions

### 2.1. Synthesis and Crystallization

**Scheme 2:**
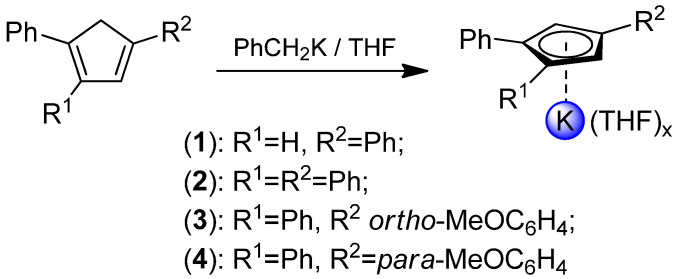
Synthesis of K(THF)_x_(Aryl_n_C_5_H_5−n_).

Synthesis of polyarylcyclopenadienyl potassium tetrahydrofuranates (KCp’(THF)_x_, **1–4**, Scheme 2) is straightforward and involves a reaction between benzyl potassium and corresponding aryl-substituted cyclopentadiene in THF. The use of benzylpotassium for the metalation of polyarylcyclopentadienes has significant advantages over other reagents, such as potassium hydride, since it allows the reaction to be carried out in a homogeneous system with 100% cyclopentadiene conversion to a target salt.

Crystals of [K(1,2,4-Ph_3_C_5_H_2_)(THF)_2_]_7_•(hexane)•(THF)_3_ (**2b**) and of [K{1,2-Ph_2_-4-(2-MeOC_6_H_4_)C_5_H_2_}(THF)] (**3**) were obtained by recrystallization from a THF/hexane mixture. Crystals of [K(1,2,4-Ph_3_C_5_H_2_)(toluene)_0.5_]•(toluene)_0.5_ (**2a**) were grown from a toluene/hexane mixture. Numerous attempts to crystallize K(1,3-Ph_2_C_5_H_3_)(THF)_x_ (**1**) and K(1,2,4-Ph_3_C_5_H_2_)(THF)_x_ (**4**) using various σ-donating ligands (THF, DME, diglyme, 18-crown-6, toluene) and mixtures thereof allowed us to obtain single crystals of [K(1,3-Ph_2_C_5_H_3_)(diglyme)] (**1a**) and [K{1,2-Ph_2_-4-(4-MeOC_6_H_4_)C_5_H_2_}(diglyme)_0.89_(THF)_0.21_]_7_•(diglyme)_0.87_ (**4a**) from a THF/diglyme/hexane mixture only.

The ^1^H and ^13^C{^1^H} NMR spectra for in-situ-generated K(1,3-Ph_2_C_5_H_3_) in DMSO_d6_ [10] have been reported earlier and revealed non-rigid behavior of the anion, namely, free rotation of Ph-groups around C_ipso-Cp_-C_ipso-Ph_ bonds. The ^1^H and ^13^C NMR spectral data for potassium derivatives **1a**, **2**, **3**, **4a** in THF_d8_ media fully confirm this idea for all studied polyarylcyclopentadienide anions (Supplementary Materials: see Figures S9–S27 for 1D and 2D spectra). The ^1^H NMR data for **2** and **3** additionally indicate that vacuum drying of these compounds leads to partial loss of coordinating THF molecules to provide formulas K(1,2,4-Ph_3_C_5_H_2_)(THF)_0.4_ and K[1,2-Ph_2_-4-(2-MeOC_6_H_4_)C_5_H_2_](THF)_0.6_, respectively.

### 2.2. Crystal Structures

#### 2.2.1. General Remarks

Although numerous structures of various alkali metal derivatives with an unsubstituted cyclopentadienyl anion and their solvatomorphs have been known for a long time and continue to be studied, at present, only a few crystal structures of alkali metal aryl-substituted cyclopentadienides are known, mainly tetra and pentaaryl-substituted ones [11,12]. Crystal structures of salts containing the anion [C_5_Ph_4_H]^−^ and various σ-donating coordinated solvent ligands are represented by six sodium [13,14,15] and one potassium [16] derivatives that are either tight or solvent-separated ion pairs. The salts of the pentarylcyclopentadienes M^+^[C_5_Aryl_5_]^−^ are represented by sodium salt of [C_5_Ph_5_]^−^ [17]; lithium derivatives of [C_5_(3,5-Alkyl_2_C_6_H_3_)_5_]^−^ [18]; lithium [19] sodium [19] and potassium [19] salts having anion [C_5_(3,5-Aryl_2_C_6_H_3_)_5_]^−^; and lithium [20], potassium [20,21] or cesium [20] salts bearing anion [C_5_(4-AlkylC_6_H_4_)_5_]^−^. The structures of the last [C_5_Aryl_5_]^−^ series are solvent-separated ion pairs mainly of complex architecture or 1D coordination polymers.

There are only a few examples of monoaryl-substituted derivatives such as lithium [22], sodium [22] and potassium [23] salts of the corresponding cyclopentadienes M^+^[C_5_PhAlkyl_4_]^−^, as well as lithium and potassium salts of [C_5_(2,6-Mes_2_C_6_H_3_)H_4_]^−^ [24]. Two structures of 1,4-diphenyl-2-R-substituted cyclopentadienide-anion are also known: potassium derivative (R = iminyl) [25] and potassium salt of the dianion [C_5_Ph_2_H_2_-(CH^t^Bu)_2_-C_5_Ph_2_H_2_]^2−^ [26]. These are the only crystal structures of alkali metal (poly)aryl-substituted cyclopentadienides that have been established up to date. The lack of crystal structures of alkali metal di- and triaryl-substituted cyclopentadienides is obvious.

**Scheme 3:**
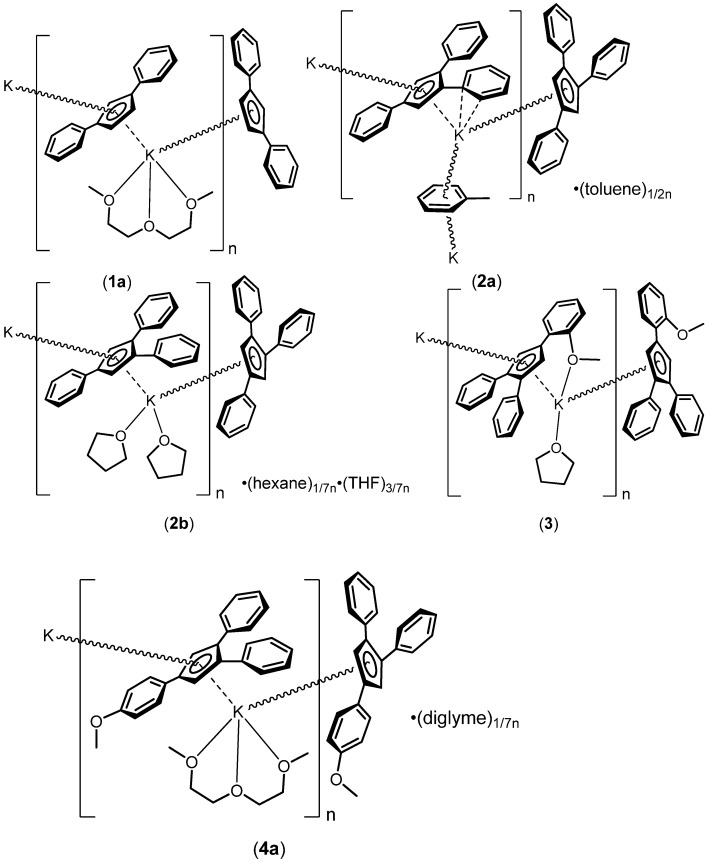
Potassium polyarylcyclopentadienide derivatives studied by X-ray diffraction analysis.

The potassium derivatives studied in this work display 1D and 2D-coordination polymer structures (**1a**, **2b**, **3**, **4a** and **2a**, correspondingly, see Scheme 3). It might be noted that in **4a** coordinated diglyme is partially replaced with THF (not shown in Scheme 3, see Section 2.2.5 below). The following description is arranged according to similarity of their crystal structures.

#### 2.2.2. Crystal Structure of [K(1,3-Ph_2_C_5_H_3_)(diglyme)], **1a**

**Figure 1:**
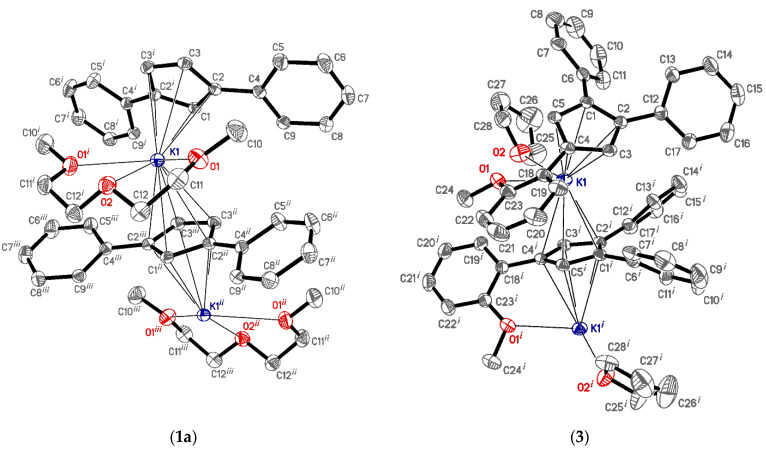
The coordination polymer structures of {[K(diglyme)(1,3-Ph_2_C_5_H_3_)}_∞_, **1a**, (**left**) and of [K(THF){1,2-Ph_2_-4-(2-MeOC_6_H_4_)C_5_H_2_}]_∞_, **3**, (**right**). Displacement ellipsoids are drawn at a 50% probability level. All H atoms are omitted for clarity.

**Figure 2:**
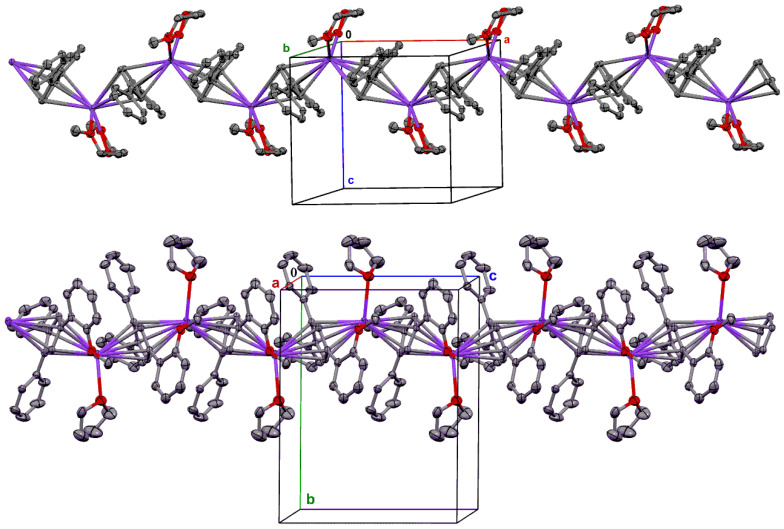
A 1D coordination polymer zigzag chain of {[K(diglyme)(1,3-Ph_2_C_5_H_3_)}_∞_, **1a**, along the *a* axis (**top**), and of [K(THF){1,2-Ph_2_-4-(2-MeOC_6_H_4_)C_5_H_2_}]_∞_, **3**, along the *c* axis (**bottom**). Displacement ellipsoids are set to a 50% probability level. H atoms are omitted.

The asymmetric unit of **1a** contains half the molecule (Z’ = ½). The other half is generated by a mirror plane (symmetry code for equivalent atoms: (i) *x*, −*y* + 1/2, *z*). Two [K(1,3-Ph_2_C_5_H_3_)(diglyme)] moieties shown in Figure 1 (left) are related by a glide plane (symmetry codes: (ii) *x* + 1/2, *y*, −*z* + 1/2; (iii) *x* + 1/2, −*y* + 1/2, −*z* + 1/2). The K^+^ cation is coordinated by two η^5^-cyclopentadienyl anions and by three oxygen atoms of a diglyme molecule, leading to the potassium coordination number (CN_K_) of 9. Therefore, the coordination compound **1a** represents a 1D coordination polymer [K(μ_2_-η^5^:η^5^-1,3-Ph_2_C_5_H_3_)(diglyme-κ^3^O,O′,O″)]_∞_ comprising tight ion pairs and exhibiting a zigzag chain structure with all K^+^ cations being in the plane parallel to *ac* (Figure 2, top). Selected K-C, K-O and C-C bond distances are given in Table 1. The average K-C_Cp_ distances are 3.141 Å for both Cp ligands. The average C_Cp_-C_Cp_ bond length is 1.417 Å. Atom C4 (C_ipso-Ph_) has a deviation of 0.1114(13) Å form the Cp plane outward from K^+^. The dihedral angle between the Cp and Ph planes (Ph-Cp rotation angle) is 16.07(8)°. Crystal structures of most *d*- and *f*-complexes bearing η^5^-1,3-Ph_2_C_5_H_3_^−^ anion (see the CSD [11]) demonstrate that at least one of two Cp-Ph dihedral angles is generally larger (up to 40.2°) than in **1a**, whereas the starting cyclopentadiene (1,4-Ph_2_C_5_H_4_ isomer) exhibits smaller angles (10.5° and 11.7°) [27]. This indicates that the structure of “unperturbed” 1,3-Ph_2_C_5_H_3_^−^ anion in **1a** is slightly affected by coordination with K^+^ and by formation of the 1D coordination polymer structure. The K1-Cp_(centroid)_ distances/normals (distances from K1 to the Cp planes) are equal within the given estimated standard deviations (ESDs): 2.9014(7) Å/2.8942(7) Å for Cp = C1, C2, C3, C3^i^, C2^i^ and 2.9005(7) Å/2.8993(7) Å for atoms C1^ii^, C2^ii^, C3^ii^, C3^iii^, C2^iii^. All K-C_Cp_ distances are similar (Table 1).

#### 2.2.3. Crystal Structure of [K(THF){1,2-Ph_2_-4-(2-MeOC_6_H_4_)C_5_H_2_}], **3**

Complex **3** contains the [K(THF){1,2-Ph_2_-4-(2-MeOC_6_H_4_)C_5_H_2_}] moiety (Z’ = 1), in which the K^+^ cation is coordinated by five C_Cp_ atoms and the O_Me_ atom of the cyclopentadienyl anion, and by the THF molecule. The K^+^ cation is additionally η^5^-coordinated by the cyclopentadyenyl ring of the neighboring moiety (Figure 1, right). Two [K(THF){1,2-Ph_2_-4-(2-MeOC_6_H_4_)C_5_H_2_}] fragments are symmetry related via a glide plane [symmetry code: (i) *x*, −*y* + 1/2, *z* + 1/2]. The coordination compound **3** forms a 1D coordination polymer [K(THF){μ_2_-η^5^:η^6^-1,2-Ph_2_-4-(2-MeOC_6_H_4_)C_5_H_2_}]_∞_ with CN_K_ = 8, which structure is similar to that of polymer **1a**: K^+^ cations are in one plane parallel to *bc* and form a zigzag chain (Figure 2, bottom). Selected K-C, K-O, C-C and C-O bond distances are presented in Table 2. The values of the Cp-Ph dihedral angles (41.09(5)° for Ph = C6..C11, 35.67(7)° for C12..C17 and 41.70(5)° for C18..C23) point out that Cp-Ph π-conjugation is substantially lost in **3**. Such large Cp-Ph dihedral angles for neighboring Ph-groups (at 1st and 2nd positions of the Cp) were observed in 1,2,4-triphenylcyclopentadienyl rare-earth complexes (see the CSD [11]), in **2a/2b** (see below) and even in different 1,2,4-triphenylcyclopentadienes bearing various substituents in Ph rings (see the CSD) due to non-valent Ph..Ph interactions.

The K1-Cp_(centroid)_ distances are 2.8340(6) Å for Cp = C1..C5 and 2.8502(8) Å for Cp = C1^i^..C5^i^. Corresponding K1-Cp_(plane)_ distances are 2.7965(7) Å and 2.7729(12) Å. Unlike in **1a**, the K-C and K1-Cp_(centroid)_ distances in **3** for Cp = C1..C5 are larger than those for Cp = C1^i^..C5^i^, which is likely due to presence of the K..OMe interaction. The C_ipso_(Ph) atoms are out of the Cp-plane: 0.141(2) Å for C6 and 0.074(2) Å C18 (both directed toward K1) and 0.208(2) Å for C12 (away from K1).

#### 2.2.4. Crystal Structure of [{K(1,2,4-Ph_3_C_5_H_2_)(THF)_2_}_7_(hexane)(THF)_3_], **2b**

**Figure 3:**
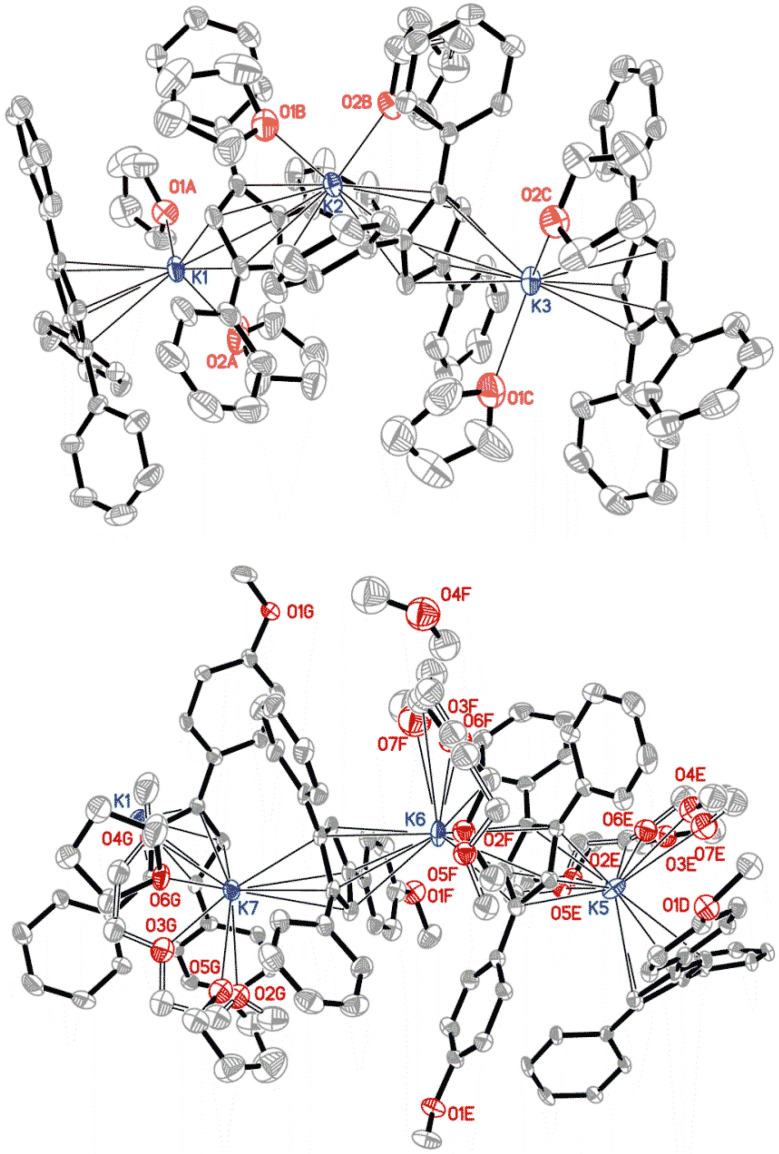
Ligand coordination modes in **2b** (**top**) and **4a** (**bottom**). Only fragments of the structures are shown. Hydrogen atoms are omitted. Displacement ellipsoids are drawn at a 30% probability level. For **4a**, minor components of disorder are shown in open solid lines.

**Figure 4:**
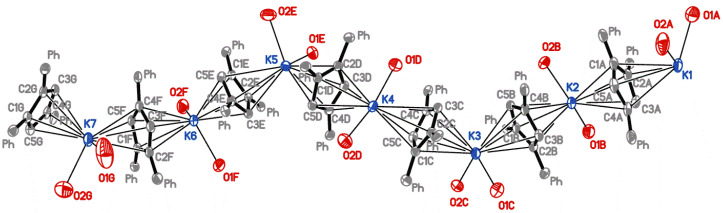
The structure of the asymmetric unit of **2b**. All hydrogen atoms and carbon atoms of Ph and THF fragments are omitted for clarity. Displacement ellipsoids are drawn at a 30% probability level.

**Figure 5:**
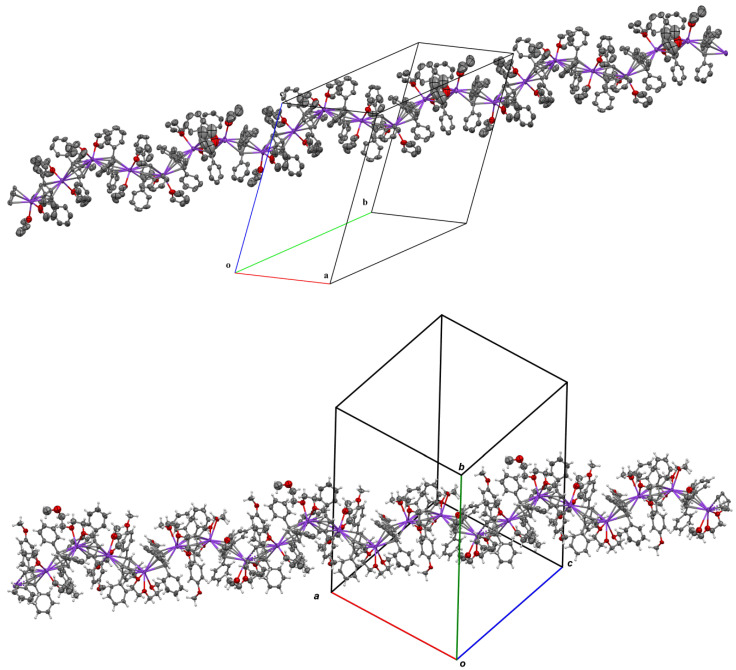
The structure of 1D coordination polymer and the unit cell for **2b** (**top**) and **4a** (**bottom**).

The asymmetric unit of complex **2b** comprises seven crystallographically independent K(1,2,4-Ph_3_C_5_H_2_)(THF)_2_ fragments (Figure 3, top) and, at least, two half-molecules of hexane, three THF molecules, which were removed by the SQUEEZE [28]. K(μ_2_-η^5^:η^5^-1,2,4-Ph_3_C_5_H_2_)(THF)_2_ (CN_K_ = 8) fragments are linked into a 1D coordination polymer, which asymmetric unit is provided in Figure 4. The chain of three asymmetric units and its orientation in a unit cell are shown in (Figure 5, top). Selected distances for seven K(1,2,4-Ph_3_C_5_H_2_)(THF)_2_ moieties are provided in Table 3.

For all seven crystallographically non-equivalent anions 1,2,4-Ph_3_C_5_H_2_^−^, all C_ipsoPh1_ atoms located at the 1st position of each Cp-anion (atoms C6A..C6G) are out of plane in one direction with corresponding deviations being in the range of 0.098(5)–0.160(6) Å, while C_ipsoPh2_ atoms at the 2nd position (atoms C12A..C12G) are out of plane in the opposite direction by 0.019(5)–0.181(5) Å. The other C_ipsoPh3_ atoms at the 4th position of Cp (atoms C12A..C12G) exhibit much smaller deviations from the Cp-plane in any of two directions by 0.002(5)–0.056(6) Å. Dihedral angles Cp-Ph (Table S1) range from 28.00(10)° to 41.73(13)° for Ph groups (Ph = C6..C11 and C12..C17) at the 1st and 2nd of the Cp ring and from 0.8(3)° to 3.91(12)° for the third Ph group (Ph = C18..C23) located at the 4th position. These facts point to high steric hindrance in a nearly unperturbed anion, caused by non-covalent repulsion between two neighboring Ph groups at positions 1 and 2, and, hence, a significant loss of the π-π Cp-Ph conjugation for the corresponding Ph groups.

#### 2.2.5. Crystal Structure of [{K(diglyme)[1,2-Ph_2_-(4-MeOC_6_H_4_)C_5_H_2_]}_6_ {K(diglyme)_0.25_(THF)_1.5_[1,2-Ph_2_-(4-MeOC_6_H_4_)C_5_H_2_]}(diglyme)_0.87_], **4a**

Regardless of crystallization in different crystal systems, crystal structures of complexes **2b** and **4a** have a very similar but more complicated structural motif. Thus, the asymmetric unit of **4a** (not shown), which is similar to that of **2b** (Figure 4) but more complicated, contains six {K(diglyme)[μ_2_-η^5^:η^5^-1,2-Ph_2_-(4-MeOC_6_H_4_)C_5_H_2_]} fragments (labeled from *A* to *F* in the CIF file) and one fragment {K(diglyme)_0.25_(THF)_1.5_[1,2-Ph_2_-(4-MeOC_6_H_4_)C_5_H_2_]} (labeled as *G*) with partial occupancies for coordinated diglyme and THF molecules. In other words, fragments *G* bearing one diglyme molecule (atoms O2G, O3G and O4G in Figure 3) occur with probability of 0.250(5), and those bearing two THF molecules (atoms O5G and O6G)—0.750(5). In nearly all fragments (*A*-*G*), diglyme exhibits the κ^3^O,O′,O″-coordination mode, but a major component of diglyme disorder in fragment *F* displays the κ^2^O,O′-coordination mode (atoms O2F, O3F and O4F Figure 3; site occupancy of 0.621(5)). Different coordination modes found in **4a** are shown in Figure 3 (bottom), using only three moieties of type {K[(diglyme)/(THF)_2_][1,2-Ph_2_-(4-MeOC_6_H_4_)C_5_H_2_]}. The moieties of **4a** are linked into a 1D coordination polymer analogous to **2b**, a chain of three crystallographically non-equivalent fragments of the 1D coordination polymer and the chain orientation in a unit cell are shown in Figure 5 (bottom). The asymmetric unit also includes a non-coordinating diglyme molecule, a partial occupancy of which was determined from a deformation electron density map at the final stage of structure refinement. Strictly speaking, Z’ = 1 for the whole asymmetric unit due to the presence of different coordinating solvents and a non-coordinating diglyme molecule. A less formal approach for description of the polymer may use Z’ = 7, since the structure has seven non-equivalent {K[(diglyme)/(THF)_2_][1,2-Ph_2_-(4-MeOC_6_H_4_)C_5_H_2_]} fragments. The selected distances in **4a** are presented in Table 4.

The steric factors in the Cp anion of **4a** are very similar to those in **2b**. Thus, all crystallographically non-equivalent anions [1,2-Ph_2_-(4-MeOC_6_H_4_)C_5_H_2_] demonstrate deviations of two atoms C_ipsoPh_ from the Cp plane in opposite directions from 0.074(9) Å to 0.227(9) Å, whereas deviations for C_ipso_ of the 4-methoxyphenyl group are significantly smaller: 0.003(9)–0.074(9) Å. The Cp-Ph dihedral angles (Table S2) are in the range of 24.3(3)° to 43.2(3)° with the exception of one Ph ring (C6..C11) in the moiety B, with a Cp-Ph angle of 17.1(3)°. The Cp-(4-MeOC_6_H_4_) dihedral angles are smaller and range from 1.9(4)° to 8.6(2)°.

It might be noted that larger steric non-covalent repulsive interactions appear in the crystal structures of the starting cyclopentadienes. Thus, 1,2,4-Ph_3_C_5_H_3_ displays high Cp-Ph angles 28.2°, 42.3° and 21.8° for all Ph groups located at positions 1, 2 and 4, correspondingly. Analogous Cp-Ph angles in a crystal structure of [1,2-Ph_2_-(4-MeOC_6_H_4_)C_5_H_3_] [29] are 34.5° and 46.0°, the Cp-(4-MeOC_6_H_4_) dihedral angle is 17.3°. In both cases, the last values shown for aryl groups at position 4 are even larger than that in complexes **2b**, **3** and **4a**.

All four structures mentioned above are 1D coordination polymers with the μ_2_-η^5^:η^5^-coordination modes for Cp ligands, regardless of Z’ for the 1D chain in a wider sense (Z’ = 0.5 for **1a**; 1 for **3**, Z’ = 7 for **2b** and **4a**). Complex **2a** has an additional type of coordination modes for potassium cation, which leads to a different structural type.

#### 2.2.6. Crystal Structures of [K(1,2,4-Ph_3_C_5_H_2_)(toluene)_0.5_]•(toluene)_0.5_, **2a**

**Figure 6:**
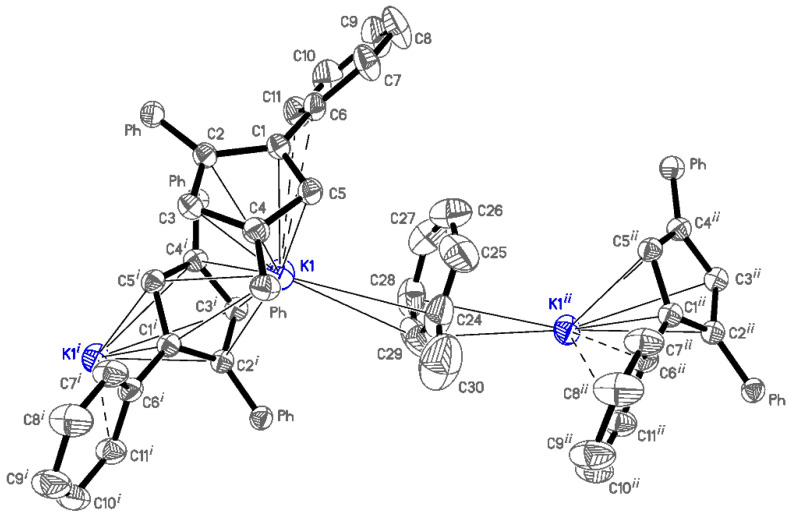
A fragment of {[K(μ_2_-η^5^:η^5^-1,2,4-Ph_3_C_5_H_2_)(μ_2_-η^2^:η^2^-toluene)_0.5_]•(toluene)0.5}_n_, **2a**. All but C_ipso_ (labeled as Ph) carbon atoms for non-interacting Ph groups and all H-atoms are omitted for clarity. The second component of the disorder for the coordinated toluene molecule and a lattice toluene molecule are not shown. Displacement ellipsoids are drawn at the 50% probability level.

**Figure 7:**
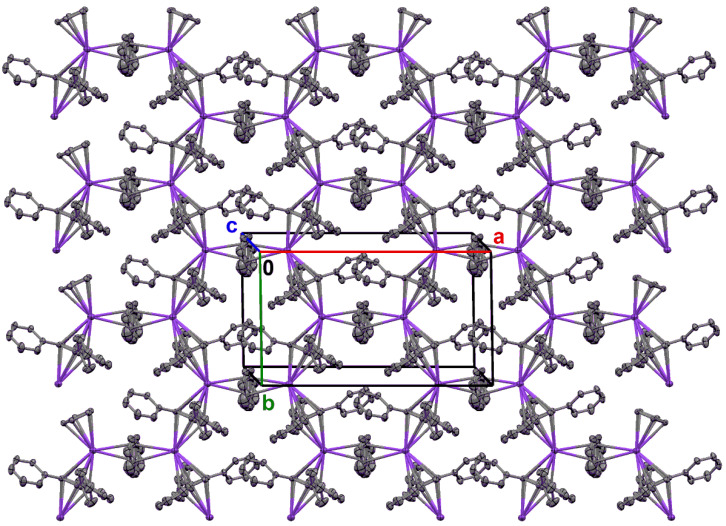
One 2D coordination polymer layer of [K(μ_2_-η^5^:η^5^-1,2,4-Ph_3_C_5_H_2_)(μ_2_-η^2^:η^2^-toluene)_0.5_]_n_ parallel to the *ab* plane in **2a**. Displacement ellipsoids are set to a 50% probability level. H atoms and non-coordinating toluene molecules are omitted.

The asymmetric unit of complex **2a** contains K^+^, [1,2,4-Ph_3_C_5_H_2_]^−^, a coordinated toluene molecule disordered over a 2-fold proper rotation axis (50% atom site occupancies) (Figure 6, Table 5) and a non-coordinating toluene molecule located at an inversion center (not shown). The composition corresponds to the formula [K(μ_2_-η^5^:η^7^-1,2,4-Ph_3_C_5_H_2_)(μ_2_-η^2^:η^2^-toluene)_0.5_]•(toluene)_0.5_. K^+^ is coordinated with two symmetry related Cp-rings: the K1-Cp_(centroid)_/K1-Cp_(plane)_ distances are 2.9185(6) Å/2.8637(7) Å for Cp = C1..C5 and 2.8169(7)Å/2.8068(7)Å for C1^i^..C5^i^ (symmetry code: (i) –*x* + 3/2, *y* – 1/2, –*z* + 3/2; a 2-fold screw axis). The lack of σ-donating solvents leads to additional contacts of K^+^ with one C_ipso-Ph_ and one C_ortho-Ph_ atoms (C6 and C11), as well with the π-system of the toluene molecule (C24, C29 or C28, C29). The K-C and selected C-C bond distances are given in Table 3. The Cp-Ph angles for neighboring Ph-groups are rather large, being of 49.52(7)° (Ph = C6..C11) and 32.59(6)° (C12..C17). The Cp-Ph angle for the 3rd Ph-group (C18..C23) is 6.64(8)°. Three contacts of K^+^ with μ_2_-bridging ligands, including one with the toluene molecule, result in formation of a 2D coordination polymer structure. The Figure 6 demonstrates a part of the structure, whose 2D layer is shown in Figure 7.

### 2.3. Luminescent Studies

**Figure 8:**
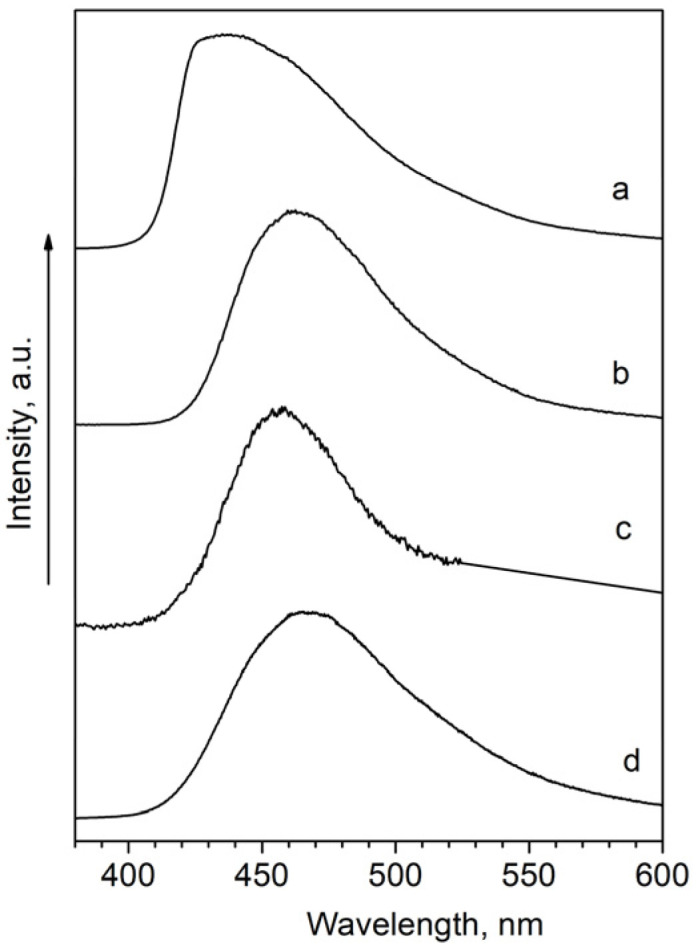
Luminescence spectra of powdered compounds **1** (a), **2b** (b), **3** (c) and **4a** (d) at 300 K, λ_exc_ = 280 nm.

All the studied compounds exhibit luminescence at 77 and 300 K. The emission bands are very wide, their maxima are ~435 nm, 460 nm, 455 nm and 465 nm for **1**, **2b**, **3** and **4a**, respectively, at 300 K (Figure 8). Compared to emission maximum of **1,** the other maxima are redshifted (**2b**, **3** and **4a**). These data are expected considering that the increase in the number of phenyl substituents leads to decrease in the energy of the emission band, which we observed earlier for lanthanide complexes with di-, tri- and tetraphenyl substituted Cp [9]. Interestingly, these maxima are relatively similar in the case of **2b**, **3** and **4a** (where Cp ring contains three phenyl substituents or two phenyl- and one methoxyphenyl substituents, respectively). The overall quantum yields of photoluminescence for compounds **1**, **2b**, **3** and **4a** are 18, 5, 3 and 1%, respectively. To understand the difference obtained in these values, it should be noted that in **1**, the dihedral angle between the Cp and Ph planes is the smallest (16.07(8)°), which reflects the coplanarity of substitutions with the Cp plane and, as a consequence, extends the length of π-system.

**Figure 9:**
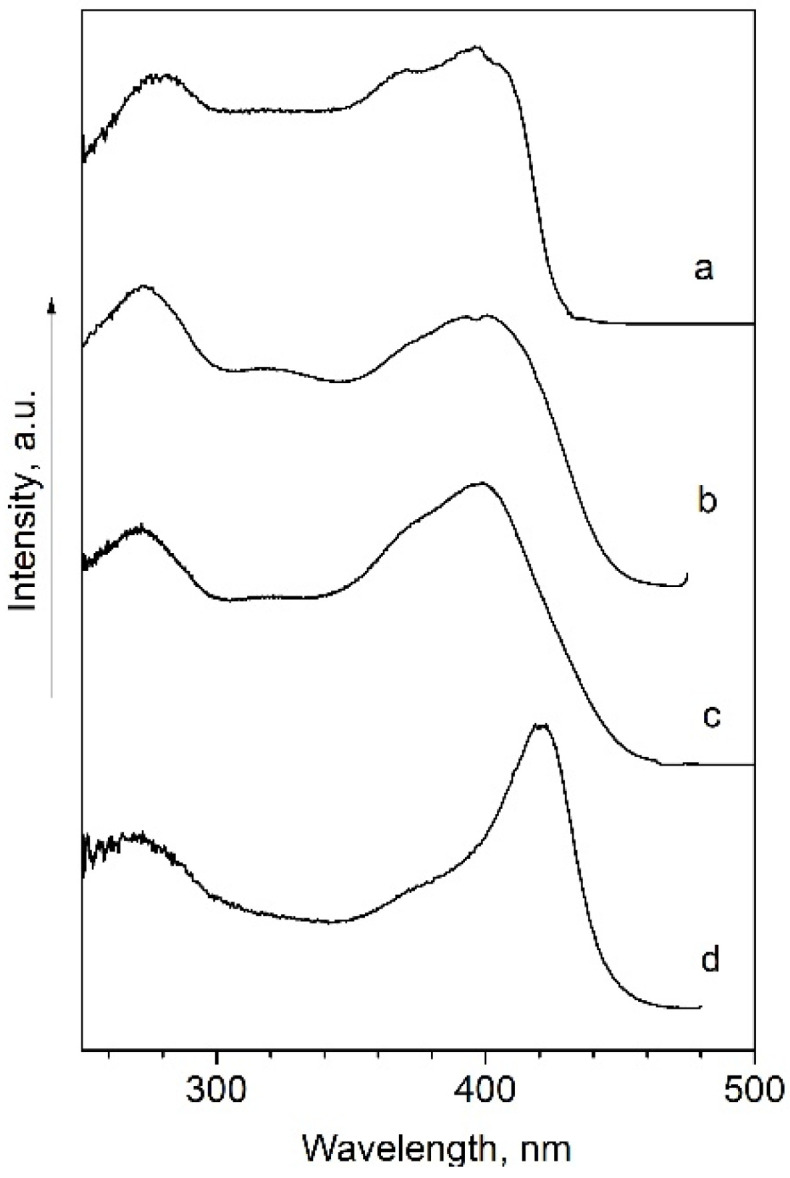
Luminescence excitation spectra of compounds **1** (a), **2b** (b), **3** (c) and **4a** (d) at 77 K.

The luminescence excitation spectra of the compounds **1**, **2b**, **3** and **4a** exhibit broad bands in the region 250–450 nm (Figure 9). Namely, a broad band centered at ~270 nm is tentatively assigned to unsubstituted Cp, while the band at ~370–415 nm is probably due to the attached phenyl rings and intraligand charge transfer (ILCT) state [9,30,31]. As it was shown earlier, the energy of the band assigned to the phenyl ring of substituted Cp depends on the number of these rings and their coplanarity with Cp. The most intense ILCT band is observed in **4a** where the dihedral angle of one Ph ring is relatively small 17.1(3)° and Cp-(4-MeOC_6_H_4_) dihedral angles range from 1.9(4)° to 8.6(2)°. These data point to high degree of coplanarity of substitutions with Cp. Thus, the presence of one phenyl with methoxy group and two unsubstituted phenyls leads to significant non-equivalence of aryl groups in this ligand and promote**s** the ILCT.

## 3. Materials and Methods

### 3.1. General Experimental Remarks

The potassium arylcyclopentadienyl complexes are extremely sensitive, even to the traces of oxygen and moisture. All of these compounds completely or significantly decompose when exposed to air for less than 1–2 s. All operations with potassium arylcyclopentadienides, including their synthesis, isolation, crystallization, preparation of samples for NMR spectroscopy, luminescence studies and X-ray diffraction analysis, were carried out inside a Specs-GB2 argon glove box. The box atmosphere contained less than 1 ppm of oxygen and water. Samples for NMR spectroscopy were prepared in J.Young NMR tubes, THF-d8 was vacuum transferred into the J.Young NMR-tube.

Samples for luminescence studies were placed in cuvettes, which were quartz tubes 4 mm in diameter, sealed at one end and equipped with a stopcock. The filled cuvettes were removed from the glove box, attached to a vacuum line and evacuated (10^−2^ torr). Then, they were sealed using a quartz-blowing torch. To prepare samples for X-ray diffraction analysis, crystals of the obtained compounds were placed in liquid paraffin in the glove box into the screw-capped vials. Liquid paraffin was distilled over sodium in high vacuum (2−5 × 10^−2^ torr) prior to use.

Tetrahydrofuran (THF) and diethyl ether (Et_2_O) were predried over NaOH and distilled from sodium/benzophenone ketyl. Hexane and THF_d-8_ (99.5 atom % D, Aldrich) were distilled from Na/K alloy. Diglyme was predried over NaOH, and then vacuum distilled from sodium. Toluene was distilled from sodium/benzophenone ketyl. BuLi (2.5M, hexane solution, Aldrich-Sigma) and potassium *tert*-pentoxide (potassium (2-methyl-2-butoxide), ~1.7M solution in toluene, Aldrich-Sigma) were used as purchased. Benzyl potassium, PhCH_2_K, was prepared according to the literature procedures [32,33]. Saturated hydrocarbon oil used for crystal preparation for X-ray studies was vacuum distilled over sodium and kept under argon in a glove box. 1,2-Diphenyl-4-(2-methoxyphenyl)cyclopenta-1,3-diene, 1,2-diphenyl-4-(4-methoxyphenyl)cyclopenta-1,3-diene, 1,2,4-triphenylcyclopenta-1,3-diene and 1,3-diphenylcyclopentadiene (as a mixture of tautomers) were obtained by the published method [29,34,35]; then, they were recrystallized from methyl *tert*-butyl ether and vacuum sublimed prior to use; see the Supporting Information (SI) for their NMR spectra (Figures S1–S8). It might be noted that initially obtained 1,3-diphenylcyclopenta-1,3-diene transforms upon purification into its 1,4-tautomer, which has been used to prepare **2**.

^1^H, ^13^C{^1^H}, ^1^H-^1^H COSY, ^1^H-^13^C HSQC, ^1^H-^13^C HMBC NMR spectra were recorded with Bruker Avance-III -600 (600 MHz for ^1^H and 150.9 MHz for ^13^C) spectrometer. The spectra are reported in SI with signal assignments.

### 3.2. Synthesis and Crystallization

[K(diglyme)(1,3-Ph_3_C_5_H_2_)] (**1a**) Benzylpotassium (0.53 mmol 69 mg) in 5 mL of THF was added to the solution of 1,4-diphenylcyclopenta-1,3-diene (0.50 mmol, 110 mg) in 10 mL of THF. The resulting solution was stirred for 30 min and then evaporated by half of the volume in vacuum; 2 mL of diglyme was added. Then, hexane (30 mL) was carefully layered on top of the formed solution. After 1 week, colorless crystals were obtained, which were removed from the solution and dried in vacuum to give 146 mg (75%) of **1a**. ^1^H NMR (600 MHz, THF_d8_) δ: 7.34 (4H, d, H*_ortho_*, ^1^J_CH_ = 154.5 Hz, ^3^J_HH_ = 7.7 Hz), 7.02 (4H, t, H*_meta_*, ^1^J_CH_ = 153.9 Hz), 6.68 (2H, t, H*_para_*, ^1^J_CH_ = 157.5Hz, ^3^J_HH_ = 7.2 Hz), 6.39 (1H, t, H2-*Cp*, ^1^J_CH_ = 155.1 Hz, ^4^J_HH_ = 2.2 Hz), 6.02 (2H, d, H4,5-*Cp*, ^1^J_CH_ = 156.4 Hz, ^4^J_HH_ = 2.2 Hz), 3.48 (4H, t, OC***H_2_***CH_2_OCH_3_), 3.39 (4H, t, OCH_2_C***H_2_***OCH_3_), 3.23 (6H, s, OCH_2_CH_2_OC***H_3_***). ^13^C{^1^H} NMR (151MHz, THF_d8_) δ: 142.86 (C*_ipso_*-Ph), 128.66 (C*_meta_*), 123.80 (C*_ortho_*), 123.42 (C*_ipso_*-Cp), 121.22 (C*_para_*), 106.73 (C4,C5-Cp), 101.58 (C2-Cp), 72.94 (OCH_2_***C***H_2_OCH_3_), 71.36 (O***C***H_2_CH_2_OCH_3_), 59.05 (OCH_2_CH_2_O***C***H_3_). For the ^1^H, ^13^C{^1^H} and ^1^H-^13^C gHSQC spectra please refer to Figures S9–S12 in SI.

[K(THF)(1,2,4-Ph_3_C_5_H_2_)] (**2**) A solution of the benzylpotassium (0.53 mmol 69 mg) in 5 mL of THF was added to the solution of 147 mg (0.50 mmol) of 1,2,4-triphenylcyclopenta-1,3-diene in 10 mL of THF. The resulting solution was stirred for 60 min and then it was evaporated to dryness. The residuals washed with hexanes and dried in vacuum The yield of colorless microcrystals was 200 mg (98%). ^1^H NMR (600 MHz, THF_d8_) δ: 7.45 (2H, d, H*_ortho_*_-Ph1_, ^1^J_CH_ = 154.5 Hz, ^3^J_HH_ = 7.7 Hz), 7.20 (4H, d, H*_ortho_*_-Ph2_, ^1^J_CH_ = 156.2 Hz, ^3^J_HH_ = 7.7 Hz), 7.10 (2H, t, H*_meta_*_-Ph1_, ^1^J_CH_ = 154.7 Hz), 6.97 (4H, t, H*_meta_*_-Ph2_, ^1^J_CH_ = 154.5 Hz), 6.81–6.74 (3H, m, H*_para_*_-Ph2_ + H*_para_*_-Ph1_; ^1^J_CH_ = 157.0 Hz and ^3^J_HH_ = 7.3 Hz for H*_para_*_-Ph2_; ^1^J_CH_ = 157.7 Hz and ^3^J_HH_ = 7.2 Hz for H*_para_*_-Ph1_), 6.25 (2H, s, H-*Cp*, ^1^J_CH_ = 156.2 Hz), 3.63–3.60 (m, -CH_2_-C***H_2_***-O_THF_), 1.79–1.76 (m, -C***H_2_***-CH_2_-O_THF_). ^13^C{^1^H} NMR (151 MHz, THF_d8_) δ: 143.49 (C*_ipso_*_-Ph2_), 142.28 (C*_ipso_*_-Ph1_), 128.86 (C*_meta_*_-Ph1_), 128.33 (C*_ortho_*_-Ph2_), 128.23 (C*_meta_*_-Ph2_), 123.68 (C*_ortho_*_-Ph1_), 122.86 (C*_ipso_*_-Cp1_), 122.48 (C*_para_*_-Ph2_), 122.38 (C*_ipso_*_-Cp2_), 121.67 (C*_para_*_-Ph1_), 108.32 (C*_H_*-Cp), 68.39 (THF, CH_2_-***C***H_2_-O), 26.54 (THF, ***C***H_2_-CH_2_-O). The ^1^H, ^1^H-^1^H COSY, ^13^C{^1^H}, ^13^C, ^1^H-^13^C gHSQC and ^1^H-^13^C HMBC spectra are presented in Figures S13–S20. X-ray quality crystals of **2a** and **2b** were obtained by crystallization from toluene (**2a**) or THF (**2b**) solution of **2** by the slow addition of hexane.

[K(THF){1,2-Ph_2_-4-(2-MeOC_6_H_4_)C_5_H_2_}] (**3**) Using the similar procedure to the synthesis of **1a**, except for the addition of diglyme, 162 mg (0.50 mmol) of 1,2-diphenyl-4-(2-methoxyphenyl)cyclopenta-1,3-diene and 69 mg (0.53 mmol) of benzylpotassium resulted in 156 mg (72%) of **3**. ^1^H NMR (600 MHz, THF_d8_) δ: 7.41 (1H, dd, *H-6*), 7.23 (4H, d, H*_ortho_*), 6.96 (4H, t, H*_meta_*), 6.74–6.82 (5H, m, H-3 + H-4 + H-5 + H*_para_*), 6.30 (2H, s, H-*Cp*), 3.69 (3H, s, O-C***H_3_***), 3.63–3.61 (m, -CH_2_-C***H_2_***-O_THF_), 1.79–1.76 (m, -C***H_2_***-CH_2_-O_THF_). ^13^C{^1^H} NMR (151 MHz, THF_d8_) δ: 156.75, 144.00, 132.49, 128.41 (C*_ortho_*), 128.17 (C*_meta_*+C-6), 122.90, 122.13, 121.68, 121.40, 119.44, 112.30, 111.87 (*C_H_-*Cp), 68.39 (THF, CH_2_-***C***H_2_-O), 55.70 (-O***C***H_3_), 26.54 (THF, ***C***H_2_-CH_2_-O). The ^1^H, ^1^H-^1^H COSY, ^13^C{^1^H} and ^13^C spectra are presented in Figures S21–S24.

[K(diglyme)_1.1_(THF)_0.1_{1,2-Ph_2_-(4-MeOC_6_H_4_)C_5_H_2_}] (**4a**) was prepared according to the synthesis of **1a**, using 69 mg (0.53 mmol) of benzylpotassium, 162 mg (0.50 mmol) of 1,2-diphenyl-4-(4-methoxyphenyl)cyclopenta-1,3-diene. After 1 week, colorless crystals were grown, which were removed from the solution for X-ray studies. The remaining microcrystals were filtered off and dried in vacuum. The yield of **4a** was 185 mg (71%). yield. ^1^H NMR (600 MHz, THF_d8_) δ: 7.39 (2H, d, H*_ortho_*_-Ph-OMe_), 7.28 (4H, d, H*_ortho_*_-Ph_), 6.99 (4H, t, H*_meta_*_-Ph_), 6.76 (2H, t, H*_para_*_-Ph_), 6.70 (2H, d, H*_meta_*_-Ph-OMe_), 6.24 (2H, s, H-*Cp*), 3.70 (3H, s, -OCH_3_), 3.63-3.61 (m, -CH_2_-C***H_2_***-O_THF_), 3.51 (t, OC***H_2_***CH_2_OCH_3_), 3.42 (t, OCH_2_C***H_2_***OCH_3_), 3.25 (s, OCH_2_CH_2_OC***H_3_***), 1.78-1.76 (m, -C***H_2_***-CH_2_-O_THF_). ^13^C{^1^H} NMR (151 MHz, THF_d8_) δ: 156.30, 144.15, 136.03, 128.24, 128.19, 124.36, 123.13, 122.14, 122.03, 114.56, 109.96, 107.87, 73.03 (diglyme), 71.47 (diglyme), 68.07 (THF), 59.07 (diglyme), 55.57, 25.95 (THF). The ^1^H, ^13^C{^1^H} and ^1^H-^13^C gHSQC spectra are presented in Figures S25–S27. The signal assignment for H-atoms is given.

### 3.3. X-ray Structure Determination

Experimental intensities of reflections were measured on a *Bruker SMART APEX II* platform, using graphite-monochromatized Mo-Kα radiation (λ = 0.71073 Å) in an ω-scan mode. The collected data were integrated with the *SAINT* program [36]. Absorption corrections based on measurements of equivalent reflections were carried out by *SADABS* (multi-scan methods) [37]. The structures were solved by direct methods with the *SHELXT* program [38] and refined by full matrix least-squares on *F*^2^ with *SHELXL* [39]. Positions of all non-H atoms were found from electron difference density maps and refined with individual anisotropic displacement parameters. Positions and individual isotropic displacement parameters for phenyl and cyclopentadienyl H-atoms in **1** and **3** were refined to obtain better quality crystallographic models. The other H-atoms in **1** and **3** and all H-atoms in **2a**, **2b** and **4a** were positioned geometrically and refined as riding atoms with relative isotropic displacement parameters. A rotating group model was applied for methyl groups. The *SHELXTL* program suite [38] and the *Mercury* program [40] were used for molecular graphics. Since the presence of poorly resolved disordered lattice solvent molecules along with a rather large volume of the unit cell in **2b** resulted in a poor crystallographic model; therefore, the non-coordinating molecules were removed by the SQUEEZE method [28] implemented in the PLATON program [41], which substantially improved the model. However, insufficient residual electron density did not allow us to resolve disorder of O- and C-atoms for some of coordinated THF molecules, resulting in level B alerts.

Crystal data, data collection and structure refinement details for **1a**, **2a**, **2b**, **3** and **4b** are summarized in Table 6. Selected bond distances and angles, as well as more detailed X-ray data refinement, are presented in the Supplementary Materials for this paper. The structures have been deposited at the Cambridge Crystallographic Data Center with the reference CCDC numbers 2206799–2206803, and they also contain the supplementary crystallographic data. These data can be obtained free of charge from the CCDC via http://www.ccdc.cam.ac.uk/data_request/cif (accessed on 11th August 2022).

### 3.4. Optical Measurements

All samples studied were solid stated. Steady-state luminescence and excitation measurements in the visible region were performed with Fluorolog FL 3-22 spectrometer from Horiba-Jobin-Yvon-Spex which has a 450 W xenon lamp as the excitation source and R-928 photomultiplier at 77 and 300 K. The quantum yield measurements were carried out on solid samples with a Spectralone-covered G8 integration sphere (GMP SA, Fällanden, Switzerland), according to the absolute method of Wrighton [42,43,44]. Each sample was measured several times under slightly different experimental conditions. The estimated error for quantum yields is ±10%.

## 4. Conclusions

The main result of the work is the demonstration of the synthetic availability of potassium cyclopentadienyl derivatives, which are convenient starting compounds for the preparation of arylcyclopentadienyl complexes of f- and d-elements, as well as determination and interpretation of their structures. These results open the way to well-defined organopotassium precursors for organometallic synthesis. The obtained data on the photophysical properties of these compounds can be useful in a comparative analysis of the photophysical properties of arylcyclopentadienyl complexes of transition and rare earth metals. Indeed, the presented results shed light on the influence of the degree coplanarity of substituents with Cp plane on the luminescence efficiency and allow the role of intra ligand charge transfer states in the energy transfer process to be highlighted.

## Figures and Tables

**Table 1:** Selected bond lengths (Å) for **1a**.

K1—O1	2.7658(9)	K1—C1 ^ii^	3.1134(14)	C2—C4	1.4651(14)
K1—O2	2.9706(12)	K1—C2 ^ii^	3.1290(10)	C3—C3 ^i^	1.400(2)
K1—C1	3.0546(14)	K1—C3 ^ii^	3.1661(11)	C4—C9	1.4026(15)
K1—C2	3.1252(10)	C1—C2	1.4175(12)		
K1—C3	3.2004(11)	C2—C3	1.4241(14)		

Symmetry codes: (i) *x*, −*y* + 1/2, *z*; (ii) *x* + 1/2, *y*, −*z* + 1/2.

**Table 2:** Selected bond lengths (Å) for **3**.

K1—C1	3.0910(12)	K1—C1*i*	2.9694(12)	C1—C2	1.4332(17)
K1—C2	3.2552(12)	K1—C2 ^i^	2.9747(12)	C1—C5	1.4176(17)
K1—C3	3.1708(13)	K1—C3 ^i^	3.0513(12)	C2—C3	1.4130(17)
K1—C4	2.9617(12)	K1—C4 ^i^	3.1067(12)	C3—C4	1.4154(18)
K1—C5	2.9112(12)	K1—C5 ^i^	3.0385(12)	C4—C5	1.4204(17)
K1—O1	2.9403(11)	O1—C23	1.3791(18)	C1—C6	1.4756(17)
K1—O2	2.7006(13)	O1—C24	1.4279(17)	C2—C12	1.4722(17)
				C4—C18	1.4737(17)

Symmetry code: (i) *x*, −*y* + 1/2, *z* + 1/2.

**Table 3:** Selected distances and distance ranges (Å) in **2b**.

	K-O1	K-O2	K-C_Cp1_	K-Cp1_centroid_	K-C_Cp2_	K-Cp2_centroid_
K1	2.681(3)	2.689(3)	3.032(3)–3.113(3) *	2.8366(15) *	3.063(3)–3.142(3)	2.8536(15)
K2	2.702(3)	2.685(3)	2.995(3)–3.110(3)	2.8092(15)	2.965(3)–3.201(3)	2.8328(15)
K3	2.687(3)	2.721(2)	2.988(3)–3.156(3)	2.8292(15)	3.008(3)–3.142(3)	2.8309(14)
K4	2.692(2)	2.693(3)	2.988(3)–3.131(3)	2.8121(14)	2.998(3)–3.138(3)	2.8173(15)
K5	2.721(3)	2.674(3)	3.017(3)–3.151(3)	2.8272(14)	2.945(3)–3.214(3)	2.8484(14)
K6	2.677(3)	2.744(3)	2.989(3)–3.207(3)	2.8539(14)	2.997(3)–3.126(3)	2.8191(15)
K7	2.753(5)	2.677(4)	3.048(3)–3.137(4)	2.8451(16)	3.064(3)–3.153(3)	2.8710(15)

* The symmetry code +*x* − 1, +*y* − 1, +*z*.

**Table 4:** Selected distances and distance ranges (Å) in **4a**.

	K-O	K-C_Cp1_	K-Cp1_centroid_	K-C_Cp2_	K-Cp2_centroid_
K1	2.688(11)–3.379(13)	3.053(6)–3.210(5) *	2.889(3) *	3.063(6)–3.117(5)	2.852(3)
K2	2.678(4)–3.037(4)	3.058(5)–3.151(5)	2.858(3)	3.085(5)–3.157(5)	2.844(2)
K3	2.617(16)–3.106(18)	3.012(5)–3.245(5)	2.884(3)	3.066(5)–3.181(5)	2.862(3)
K4	2.704(4)–3.060(4)	3.056(5)–3.177(5)	2.872(2)	3.076(6)–3.133(5)	2.867(3)
K5	2.599(11)–2.989(13)	2.959(6)–3.295(5)	2.900(3)	3.064(6)–3.296(6)	2.947(3)
K6	2.566(7)–3.118(16)	2.984(6)–3.136(5)	2.814(3)	3.051(6)–3.203(6)	2.862(3)
K7	2.667(11)–3.23(2)	2.989(5)–3.183(6)	2.849(3)	3.040(5)–3.123(6)	2.831(3)

* The symmetry code *x* − 1, *y*, *z* + 1.

**Table 5:** Selected bond lengths and short contact distances (Å) for **2a**.

K1–C1	2.9585(13)	K1–C1 ^i^	2.9761(13)	C1–C2	1.4304(16)
K1–C2	3.1991(12)	K1–C2 ^i^	3.0151(13)	C1–C5	1.4129(17)
K1–C3	3.3569(13)	K1–C3 ^i^	3.1192(13)	C2–C3	1.4083(17)
K1–C4	3.2543(13)	K1–C4 ^i^	3.1556(13)	C3–C4	1.4154(17)
K1–C5	3.0015(13)	K1–C5 ^i^	3.0507(13)	C4–C5	1.4192(18)
K1–C6	3.4972(14)	K1–C11	3.4750(16)	C1–C6	1.4757(18)
K1–C24	3.328(4)	K1 ^ii^–C28	3.421(8)	C2–C12	1.4720(17)
K1–C29	3.277(8)	K1 ^ii^–C29	3.434(8)	C4–C18	1.4716(18)

Symmetry codes: (i) −*x* + 3/2, *y* − 1/2, −*z* + 3/2; (ii) −*x* + 2, *y*, −*z* + 3/2.

**Table 6:** Crystal data, data collection and structure refinement.

Compound	1a	2a	2b	3	4a
Formula	C_23_H_27_KO_3_	C_30_H_25_K	C_235_H_269_K_7_O_17_	C_28_H_27_KO_2_	C_216.70_H_244.64_K_7_O_29.85_
*M*_r_	390.54	424.60	3639.19	434.59	514.36
Temperature (K)	120(2)	150(2)	150(2)	120(2)	120(2)
Crystal system	Orthorhombic	Monoclinic	Triclinic	Monoclinic	Monoclinic
Space group	*P*nma	*C*2/c	$P\bar{1}$	*P*2_1_/c	*P*2_1_/c
Unit cell dimensions					
*a* (Å)	9.9849(5)	18.1331(9)	18.4374(10)	17.0562(13)	20.1862(16)
*b* (Å)	22.9124(12)	10.1328(5)	24.5483(13)	13.6321(11)	48.268(4)
*c* (Å)	9.1867(5)	26.6768(16)	25.7246(14)	10.5027(8)	20.8018(18)
α (°)	90	90	68.2280(10)	90	90
β (°)	90	108.2070(10)	72.4000(10)	102.100(2)	102.1869(17)
γ (°)	90	90	80.0790(10)	90	90
Volume, Å^3^	2101.72(19)	4656.2(4)	10282.7(10)	2387.7(3)	19811(3)
Z	4	8	2	4	4
Calcd. density (g/cm^3^)	1.234	1.211	1.175	1.209	1.207
µ (mm^−1^)	0.272	0.242	0.210	0.244	0.221
F(000)	832	1792	3896	920	7666.6
Θ range (°)	1.78–29.00	2.33–29.99	0.90–25.05	1.93–29.00	1.31–26.00
Complentess to Θ_full_/Θ_max_	1.000/1.000	0.999/0.995	1.000/1.000	1.000/1.000	0.998/0.997
Index ranges	−13 ≤ h ≤ 13	−25 ≤ h ≤ 25	−21 ≤ h ≤ 21	−23 ≤ h ≤ 23	−21 ≤ h ≤ 24
	−31 ≤ k ≤ 31	−14 ≤ k ≤ 14	−29 ≤ k ≤ 29	−18 ≤ k ≤ 18	−59 ≤ k ≤ 59
	−12 ≤ l ≤ 12	−37 ≤ l ≤ 37	−30 ≤ l ≤ 30	−14 ≤ l ≤ 14	−25 ≤ l ≤ 25
Reflections					
measured	40173	25269	85405	48850	140087
independent [*R*_int_]	2857 [0.0346]	6764 [0.0303]	36397 [0.0374]	6342 [0.0309]	38825 [0.1016]
observed [*I* > 2σ(*I*)]	2472	5107	20230	5264	19851
Data/Parameters/Restraints	2857/155/0	6764/345/42	36,397/2153/1911	6342/345/0	38,825/825/2481
*R*1/w*R*2 [I > 2σ(I)]	0.0313/0.0806	0.0452/0.1155	0.0660/0.1851	0.0403/0.1076	0.0966/0.1915
*R*1/w*R*2 (all data)	0.0384/0.0864	0.0637/0.1267	0.1107/0.2047	0.0507/0.1171	0.1959/0.2499
GooF on *F*^2^	1.062	1.038	0.971	1.039	1.081
Δρ_max_/Δρ_min_ (e Å^−3^)	0.353/−0.198	0.366/−0.230	0.705/−0.520	0.848/−0.442	0.721/−0.566
CCDC number	2206799	2206800	2206801	2206802	2206803

## Supplementary Materials

The following supporting information can be downloaded at: https://www.mdpi.com/article/10.3390/molecules27227725/s1, Figure S1: The ^1^H NMR spectrum of 1,4-Ph_3_C_5_H_3_; Figure S2: The ^13^C{^1^H} NMR spectrum of 1,4-Ph_3_C_5_H_3_; Figure S3: The ^1^H NMR spectrum of 1,2,4-Ph_3_C_5_H_3_; Figure S4: The ^13^C{^1^H} NMR spectrum of 1,2,4-Ph_3_C_5_H_3_; Figure S5: The ^1^H NMR spectrum of 1,2-Ph_2_-4-(2-MeOC_6_H_4_)C_5_H_3_; Figure S6: The ^13^C{^1^H} NMR spectrum of 1,2-Ph_2_-4-(2-MeOC_6_H_4_)C_5_H_3_; Figure S7: The ^1^H NMR spectrum of 1,2-Ph_2_-4-(4-MeOC_6_H_4_)C_5_H_3_; Figure S8: The ^13^C{^1^H} NMR spectrum of 1,2-Ph_2_-4-(4-MeOC_6_H_4_)C_5_H_3_; Figure S9: The ^1^H NMR spectrum of [K(diglyme)(1,3-Ph_2_C_5_H_3_)], (1a), in THF_d8_ at 600 MHz and 303 K. Starred peaks are residual peaks from the deuturated solvent; Figure S10: The enlarged aromatic region for the ^1^H NMR spectrum of [K(diglyme)(1,3-Ph_2_C_5_H_3_)], (1a), in THF_d8_ at 600 MHz; Figure S11: The ^13^C{^1^H} NMR spectrum of [K(diglyme)(1,3-Ph_2_C_5_H_3_)], (1a), in THF_d8_ at 150 MHz and 303 K. Starred peaks are residual peaks from the deuturated solvent; Figure S12: The ^1^H-^13^C GHSQC NMR spectrum of [K(diglyme)(1,3-Ph_2_C_5_H_3_)], (1a), in THF_d8_ at 303 K; Figure S13: The ^1^H NMR spectrum of [K(thf)_x_(1,2,4-Ph_3_C_5_H_2_)], (2), in THF_d8_ at 600 MHz and 303 K. Starred peaks are residual peaks from the deuturated solvent; Figure S14: The enlarged aromatic region for the ^1^H NMR spectrum of [K(thf)_x_(1,2,4-Ph_3_C_5_H_2_)], (2), in THF_d8_ at 600 MHz; Figure S15: The aromatic region in the ^1^H-^1^H COSY NMR spectrum of [K(thf)_x_(1,2,4-Ph_3_C_5_H_2_)], (2), in THF_d8_; Figure S16: The ^13^C{^1^H} NMR spectrum of [K(thf)_x_(1,2,4-Ph_3_C_5_H_2_)], (2), in THF_d8_ at 150 MHz and 303 K. Starred peaks are residual peaks from the deuturated solvent; Figure S17: The enlarged region from 120 to 130 ppm for the ^13^C{^1^H} NMR spectrum of [K(thf)_x_(1,2,4-Ph_3_C_5_H_2_)], (2), in THF_d8_ at 150 MHz and 303 K; Figure S18: The ^13^C NMR spectrum of [K(thf)_x_(1,2,4-Ph_3_C_5_H_2_)], (2), in THF_d8_ at 151 MHz and 303 K; Figure S19: The ^1^H-^13^C GHSQC NMR spectrum of [K(thf)_x_(1,2,4-Ph_3_C_5_H_2_)], (2), in THF_d8_; Figure S20: The ^1^H-^13^C HMBC NMR spectrum of [K(thf)_x_(1,2,4-Ph_3_C_5_H_2_)], (2), in THF_d8_; Figure S21: The ^1^H NMR spectrum of [K(thf)_x_(1,2-Ph_2_-4-(2-MeOC_6_H_4_)C_5_H_3_)], (3), in THF_d8_ at 600MHz and 303K. Starred peaks are residual peaks from the deuturated solvent; Figure S22: The ^1^H-^1^H COSY NMR spectrum of [K(thf)_x_(1,2-Ph_2_-4-(2-MeOC_6_H_4_)C_5_H_3_)], (3), in THF_d8_; Figure S23: The ^13^C{^1^H} NMR spectrum of [K(thf)_x_(1,2-Ph_2_-4-(2-MeOC_6_H_4_)C_5_H_3_)], (3), in THF_d8_ at 150 MHz and 303 K. Starred peaks are residual peaks from the deuturated solvent; Figure S24: The ^13^C NMR spectrum of [K(thf)_x_(1,2-Ph_2_-4-(2-MeOC_6_H_4_)C_5_H_3_)], (3), in THF_d8_ at 150MHz and 303K. Starred peaks are residual peaks from the deuturated solvent; Figure S25: The ^1^H NMR spectrum of [K(thf)_x_(1,2-Ph_2_-4-(4-MeOC_6_H_4_)C_5_H_3_)], (4a), in THF_d8_ at 600 MHz and 303 K. Starred peaks are residual peaks from the deuturated solvent; Figure S26: The ^13^C{^1^H} NMR spectrum of [K(thf)_x_(1,2-Ph_2_-4-(4-MeOC_6_H_4_)C_5_H_3_)], (4), in THF_d8_ at 150 MHz and 303 K. Starred peaks are residual peaks from the deuturated solvent; Figure S27: The ^1^H-^13^C GHSQC NMR spectrum of [K(thf)_x_(1,2-Ph_2_-4-(4-MeOC_6_H_4_)C_5_H_3_)], (4a), in THF_d8_; Figure S28: The chain structure of the asymmetric unit of 2b. All H and C atoms are omitted. Cp-centroids are labeled as CPA through CPG. Displacement ellipsoids are drawn at a 30% probability level; Figure S29: A view along an imaginary axis of the 1D chain of 2b displays a helix-like structure. All H and C atoms are omitted; Cp-centroids are labeled as CPA through CPG; Displacement ellipsoids are drawn at a 30% probability level; Table S1; Cp-Ph rotation angles (°) in 2b; Table S2: Cp-Ph rotation angles (°) in 4a; Figure S30: Ligand coordination modes in 2a. A disorder of the coordinated toluene is shown. Atoms are drawn as spheres of fixed radii for clarity; Table S3: Potassium coordination numbers, CN_K_*. The structures **1a**, **2a**, **2b**, **3** and **4b** have been deposited at the Cambridge Crystallographic Data Center with the reference CCDC numbers 2206799–2206803, and they also contain the supplementary crystallographic data. These data can be obtained free of charge from the CCDC via http://www.ccdc.cam.ac.uk/data_request/cif (accessed on 11 August 2022).
