# Supplementary material for: Coordination Polymers of Polyphenyl-Substituted Potassium Cyclopentadienides
Source: Molecules. 2022 Nov 9;27(22):7725. doi: 10.3390/molecules27227725 (PMC9696914; doi:10.3390/molecules27227725)
Supplement: Supplementary file 1 [file molecules-27-07725-s001.zip › molecules-2008611-supplementary.pdf]

Electronic Supporting information

Coordination Polymers of Polyphenyl-substituted Potassium Cyclopentadienides

Pavel D. Komarov [1], Kirill P. Birin [2], Alexander A. Vinogradov [1], Evgenia A. Varaksina [1,3], Lada N. Puntus[1,4], Konstantin A. Lyssenko,[5,*] Andrei V. Churakov,[6] Ilya E. Nifant'ev,[1,5] Mikhail E. Minyaev,[1,7] Dmitrii M. Roitershtein[1,7,8,*]

[1] [a] A.V.Topchiev Institute of Petrochemical Synthesis, Russian Academy of Sciences, 29 Leninsky prospect, Moscow, 119991, Russian Federation.

[2] A.N. Frumkin Institute of Physical Chemistry and Electrochemistry, Russian Academy of Sciences, 31 Leninsky Prospect, Building 4, Moscow, 119071, Russian Federation.

[3] P.N. Lebedev Physical Institute, Russian Academy of Sciences, 53 Leninsky Prospect, Moscow 119991, Russian Federation.

[4] V.A. Kotel'nikov Institute of Radioengineering and Electronics, Russian Academy of Sciences, Fryazino, Moscow reg. 141190, Russian Federation.

[5] Chemistry Department, M.V. Lomonosov Moscow State University, 1 Leninskie Gory Str., Building 3, Moscow, 119991, Russian Federation.

[6] N.S. Kurnakov Institute of General and Inorganic Chemistry, Russian Academy of Sciences, 31 Leninsky Prospect, Moscow, 119991, Russian Federation.

[7] N.D.Zelinsky Institute of Organic Chemistry, Russian Academy of Sciences, 47 Leninsky Prospect, Moscow, 119991, Russian Federation

[8] National Research University Higher School of Economics, 20 Miasnitskaya Str., Moscow, 101000, Russian Federation.

Correspondence: klyssenko@gmail.com (K.A.L.); roiter@yandex.ru (D.M.R.)

S1. ^{1}H and $^{13}C\{^{1}H\}$ NMR spectra of polyarylcyclopentadienes.

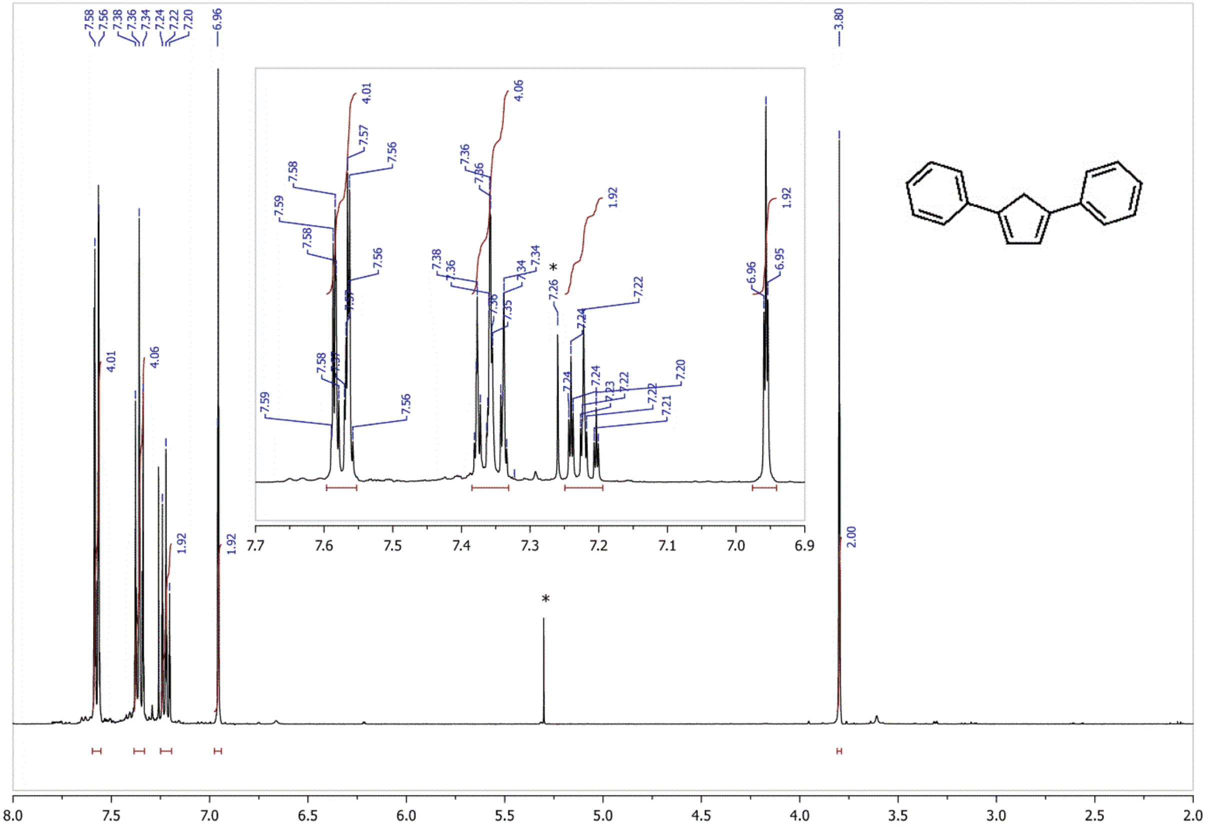

Figure S1. The ^{1}H NMR spectrum of 1,4-$Ph_3C_5H_3$.

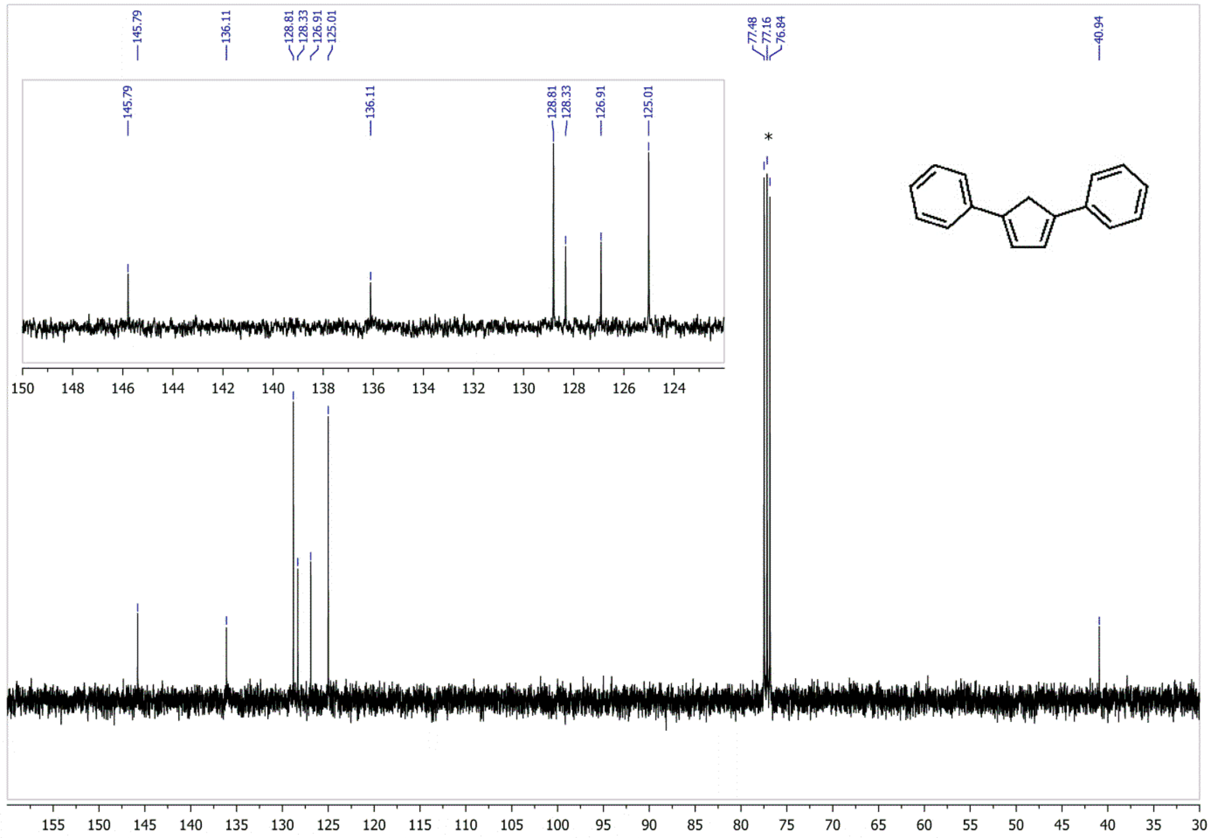

Figure S2. The $^{13}C\{^{1}H\}$ NMR spectrum of 1,4-$Ph_3C_5H_3$.

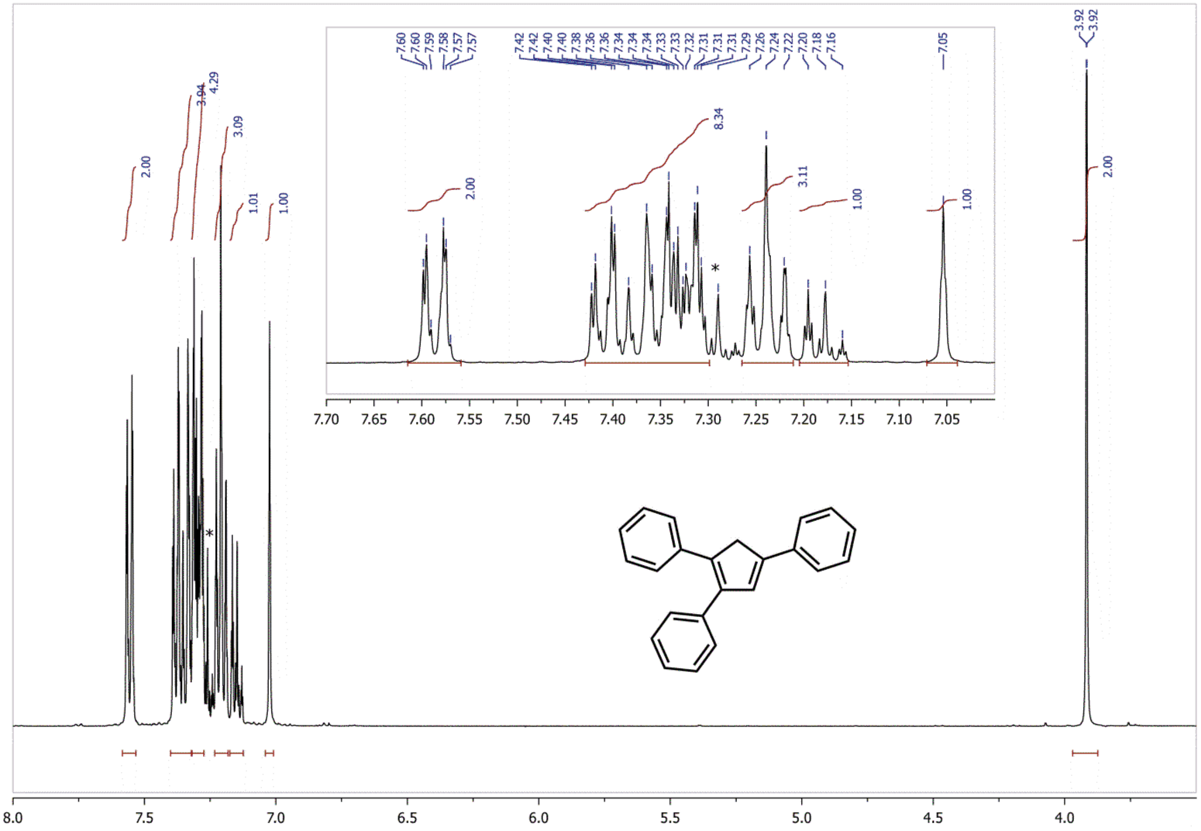

Figure S3. The ^{1}H NMR spectrum of 1,2,4-$Ph_3C_5H_3$.

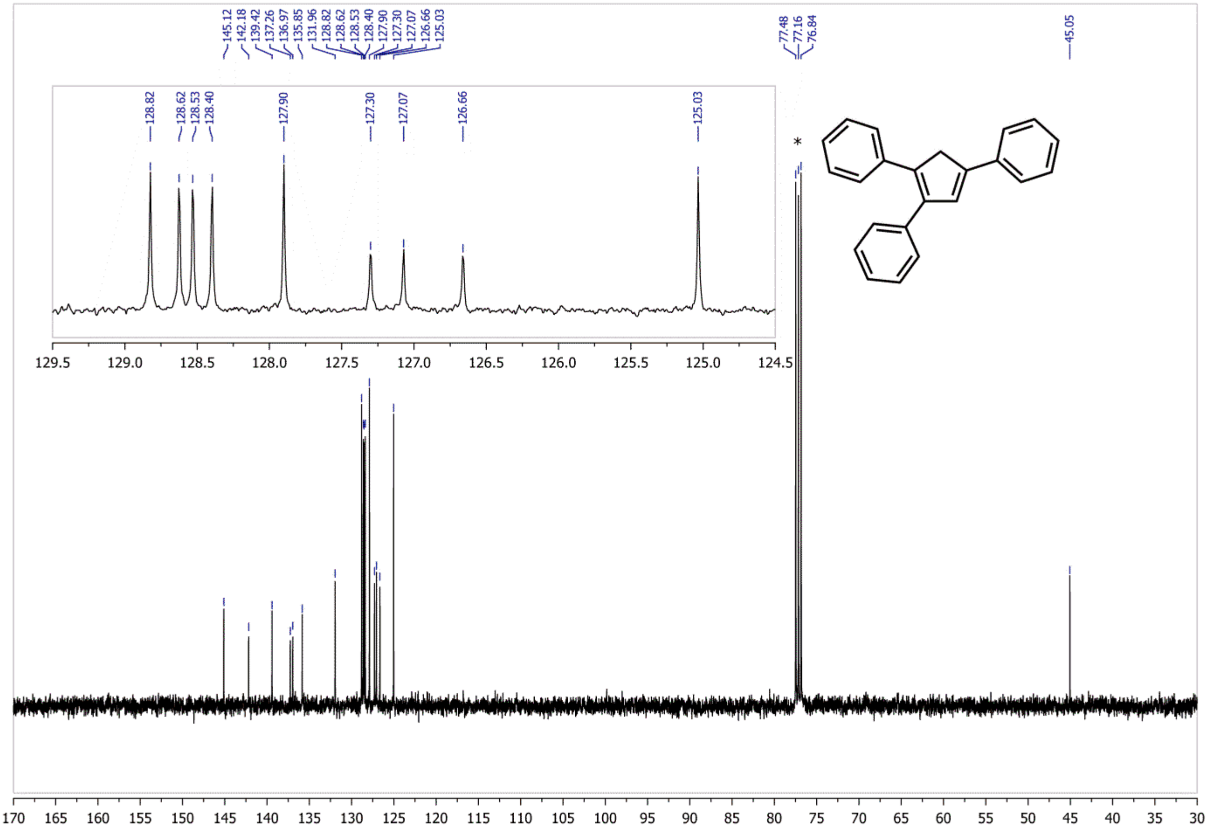

Figure S4. The $^{13}C\{^{1}H\}$ NMR spectrum of 1,2,4-$Ph_3C_5H_3$.

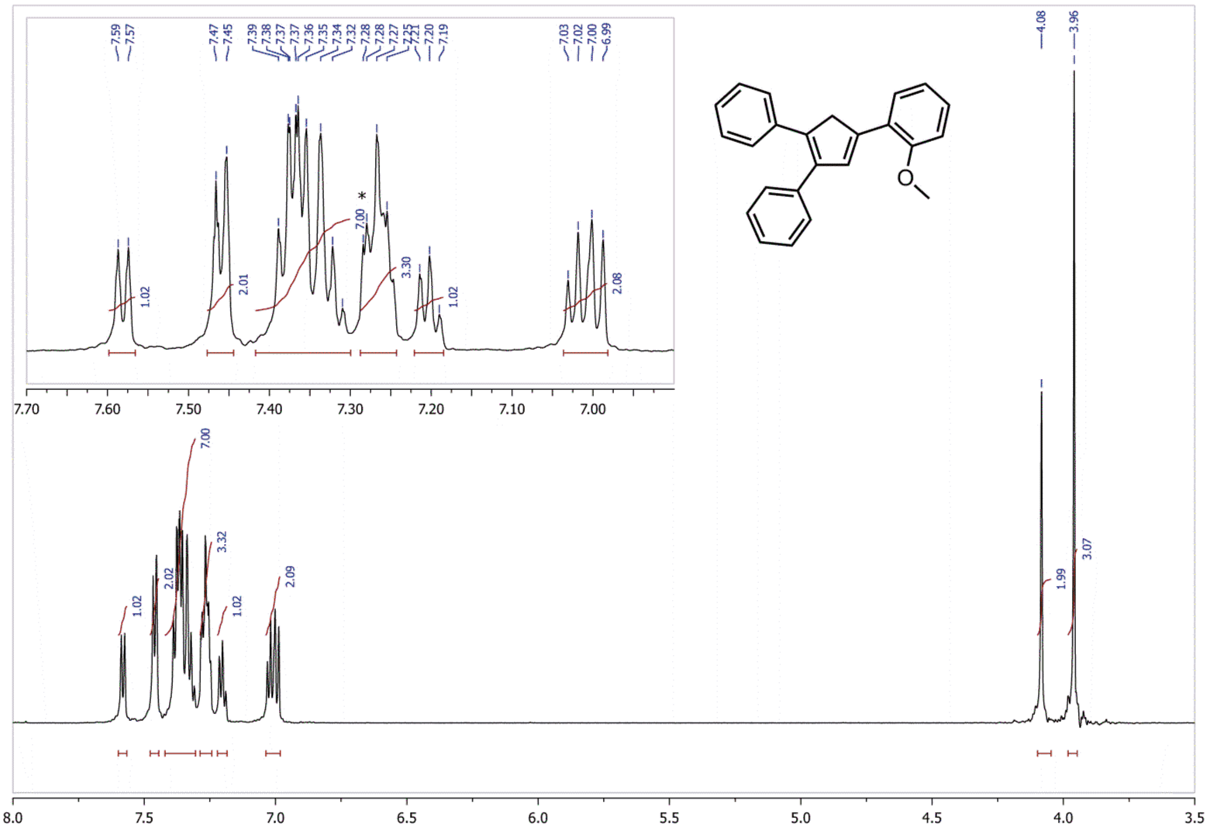

Figure S5. The ^{1}H NMR spectrum of 1,2-Ph_2-4-(2-$MeOC_6H_4$)C_5H_3.

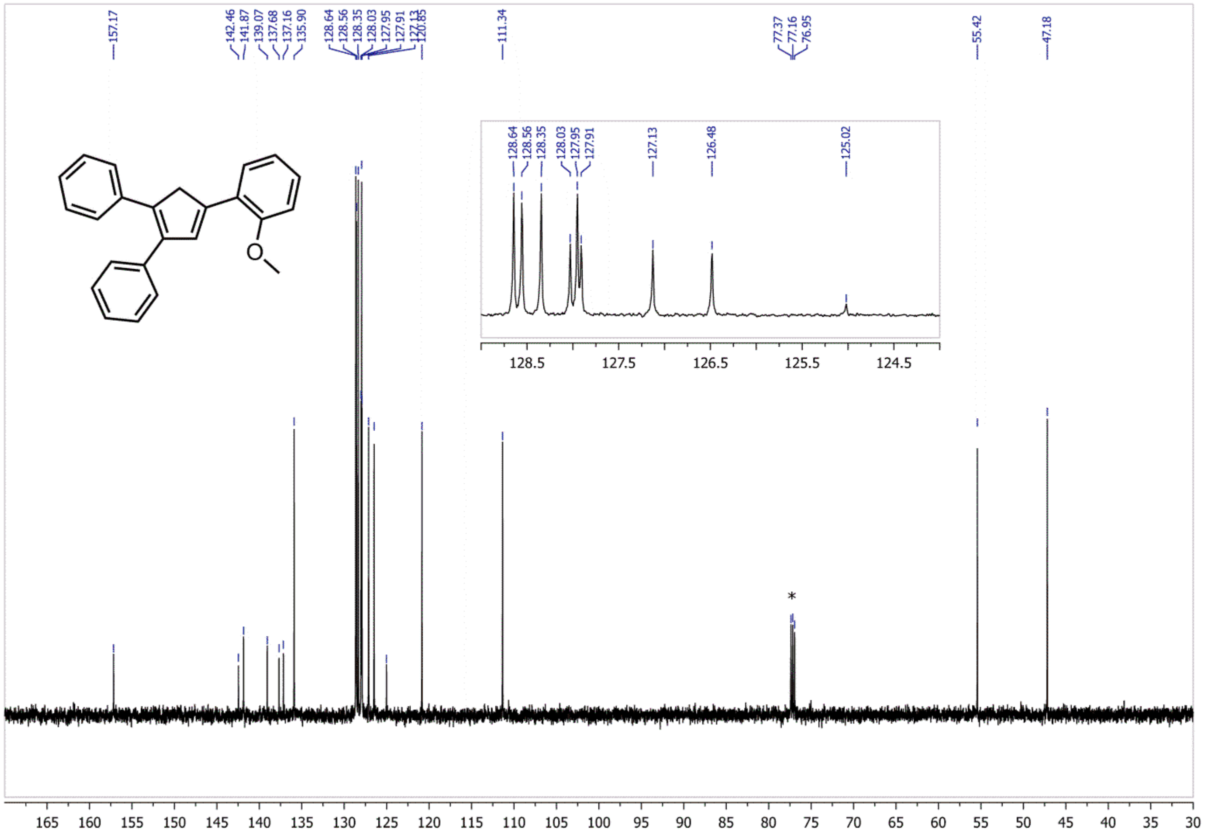

Figure S6. The $^{13}C\{^{1}H\}$ NMR spectrum of 1,2-Ph_2-4-(2-$MeOC_6H_4$)C_5H_3.

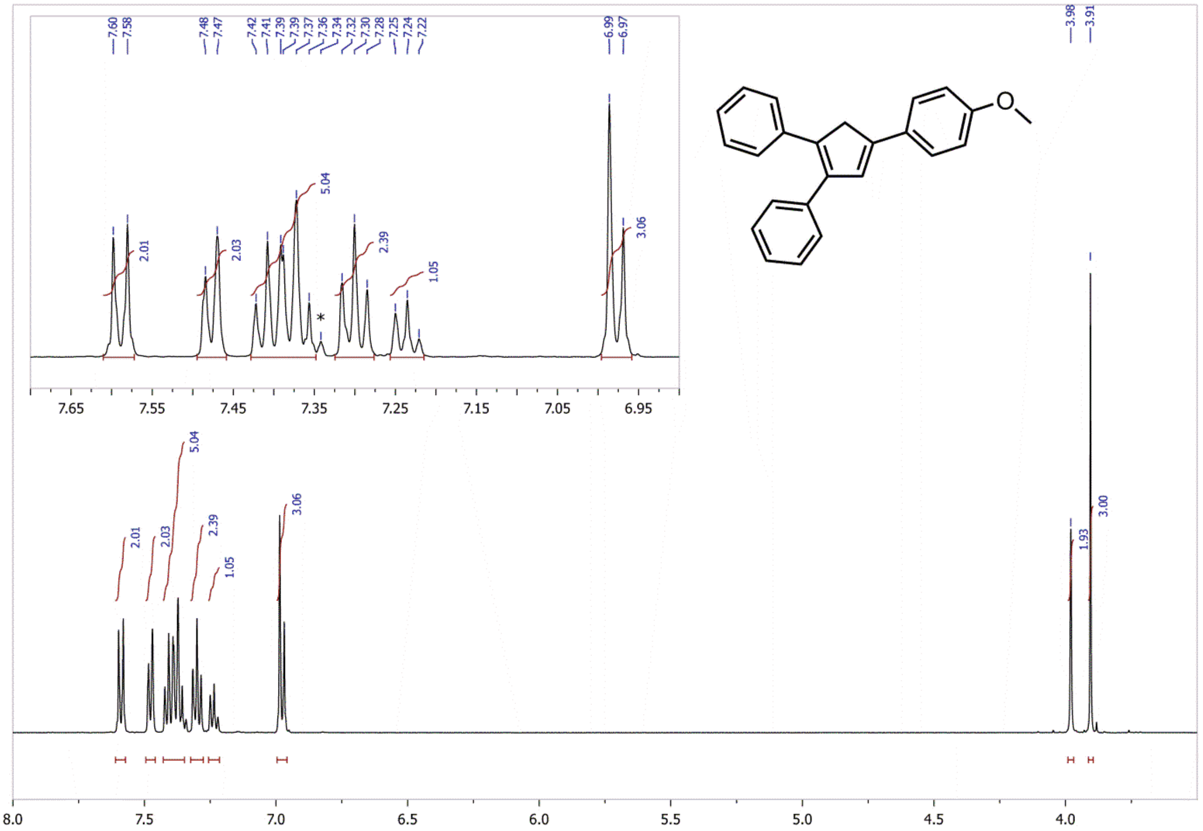

Figure S7. The ^{1}H NMR spectrum of 1,2-Ph_2-4-(4-$MeOC_6H_4$)C_5H_3.

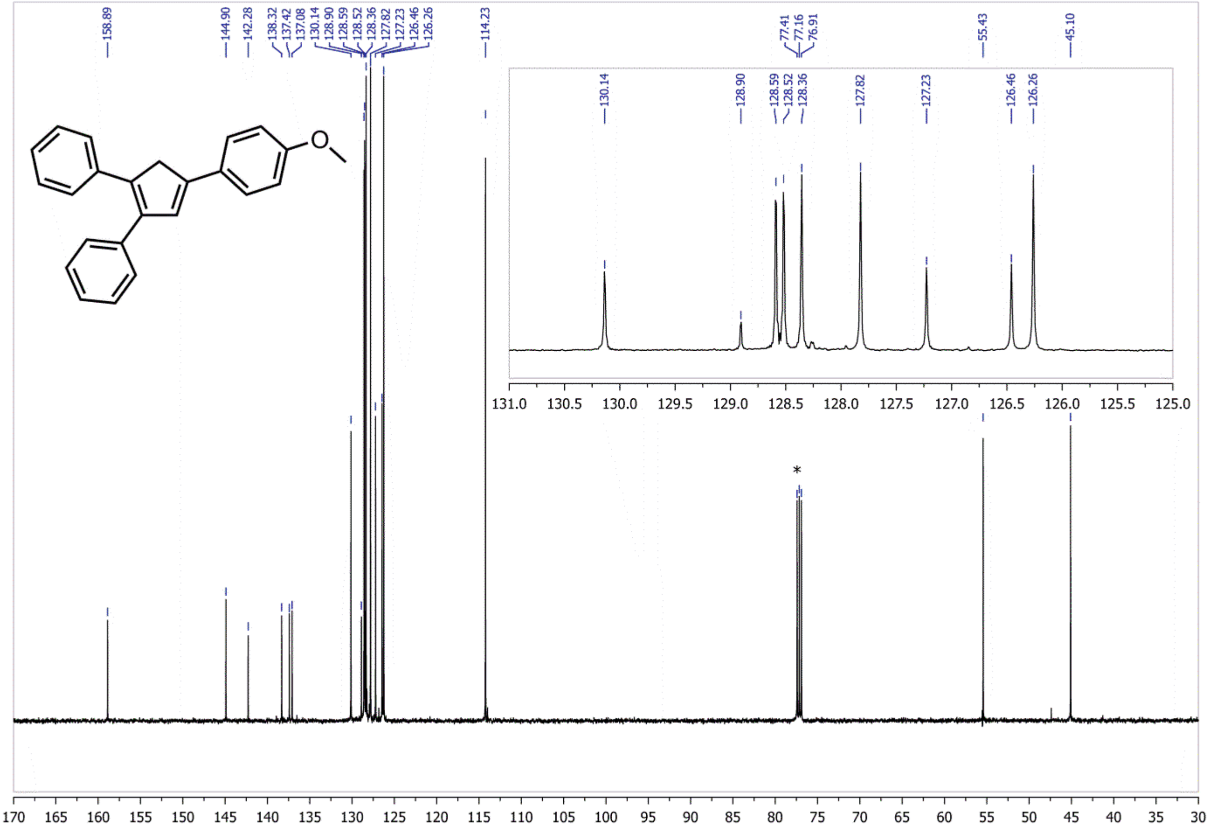

Figure S8. The $^{13}C\{^{1}H\}$ NMR spectrum of 1,2-Ph_2-4-(4-$MeOC_6H_4$)C_5H_3.

S2. NMR spectra of potassium polyarylcyclopentadienes.

S2.1. [K(diglyme)(1,3-$Ph_2C_5H_3$)], (1a).

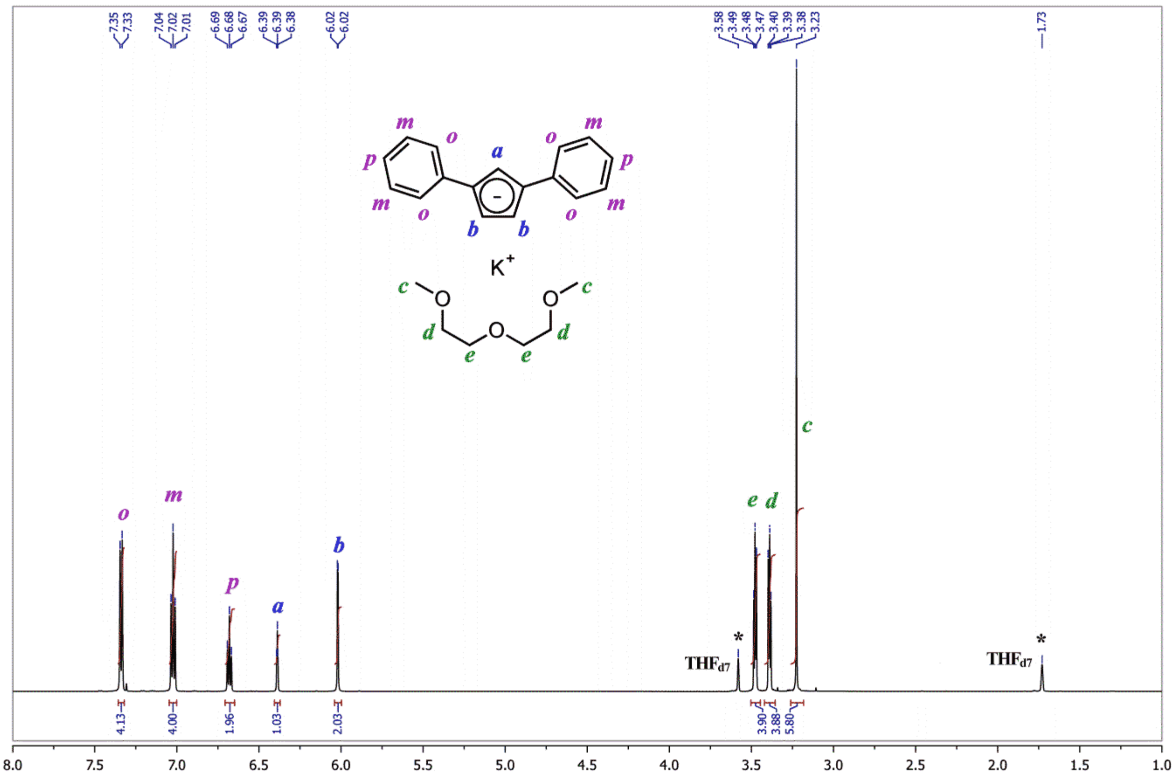

Figure S9. The ^{1}H NMR spectrum of [K(diglyme)(1,3-$Ph_2C_5H_3$)], (**1a**), in THF_{d8} at 600MHz and 303K. Starred peaks are residual peaks from the deuturated solvent.

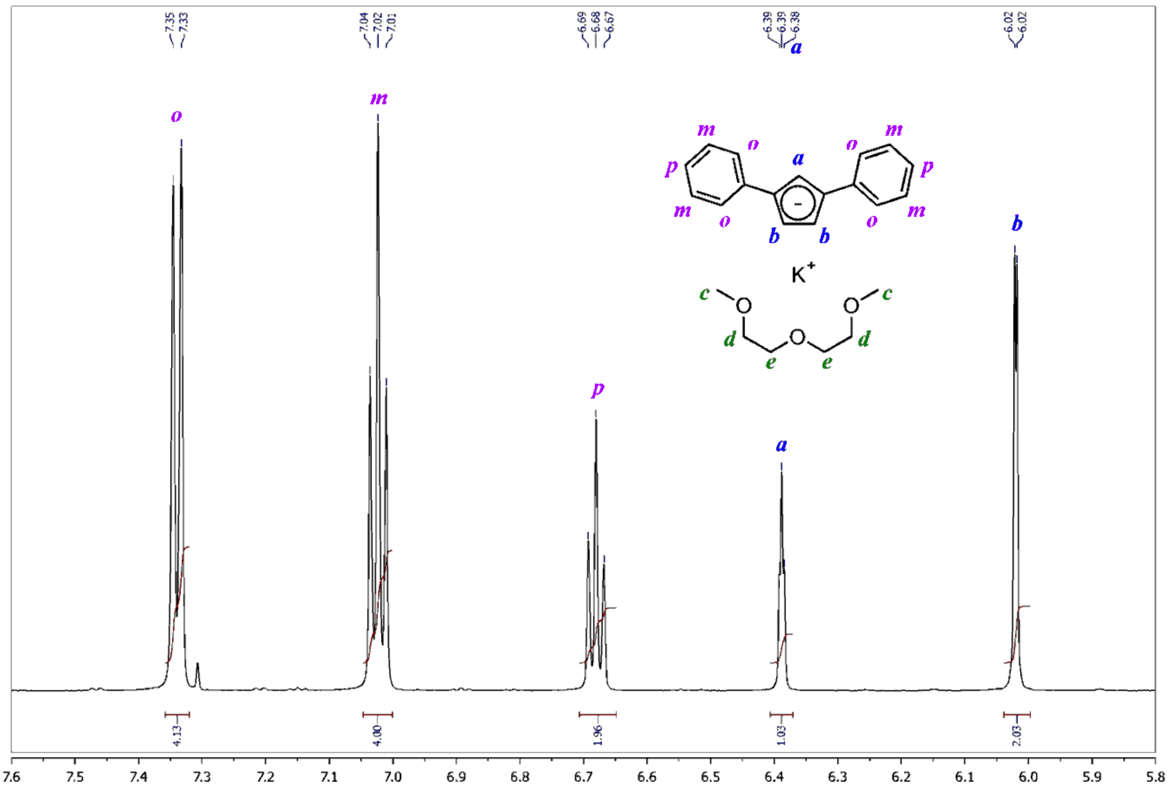

Figure S10. The enlarged aromatic region for the ^{1}H NMR spectrum of [K(diglyme)(1,3-$Ph_2C_5H_3$)], (**1a**), in THF_{d8} at 600MHz.

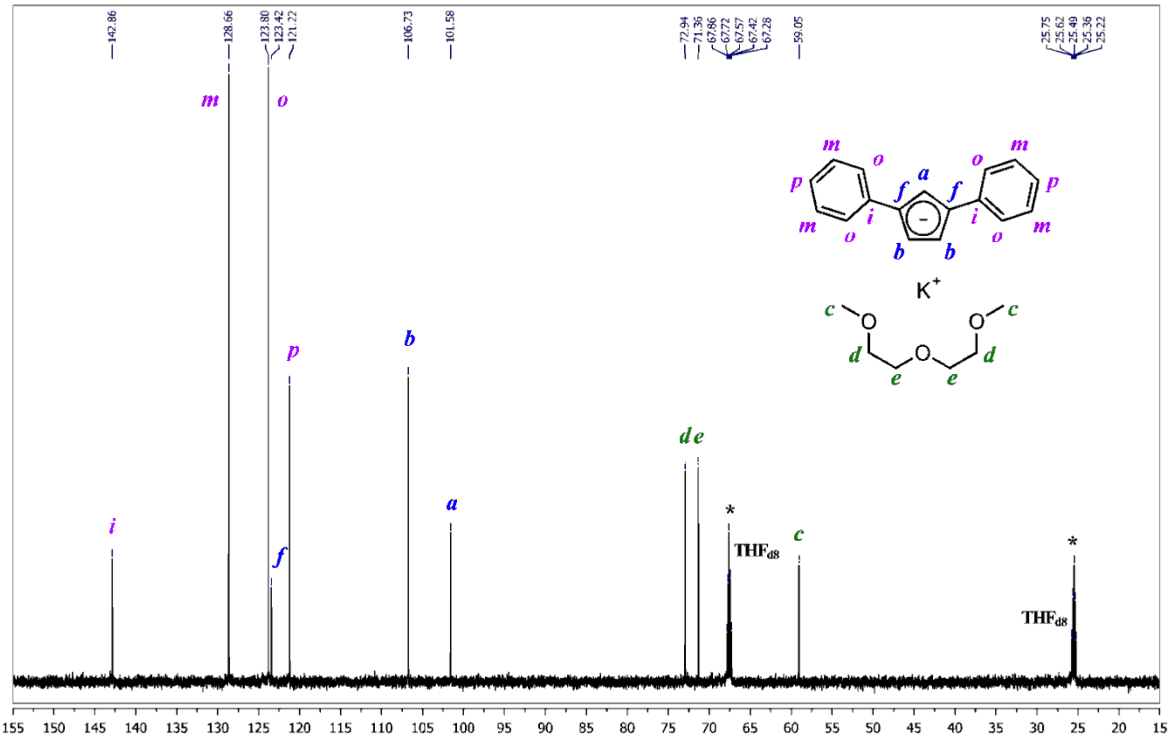

Figure S11. The $^{13}C\{^{1}H\}$ NMR spectrum of [K(diglyme)(1,3-$Ph_2C_5H_3$)], (**1a**), in THF_{d8} at 150MHz and 303K. Starred peaks are residual peaks from the deuturated solvent.

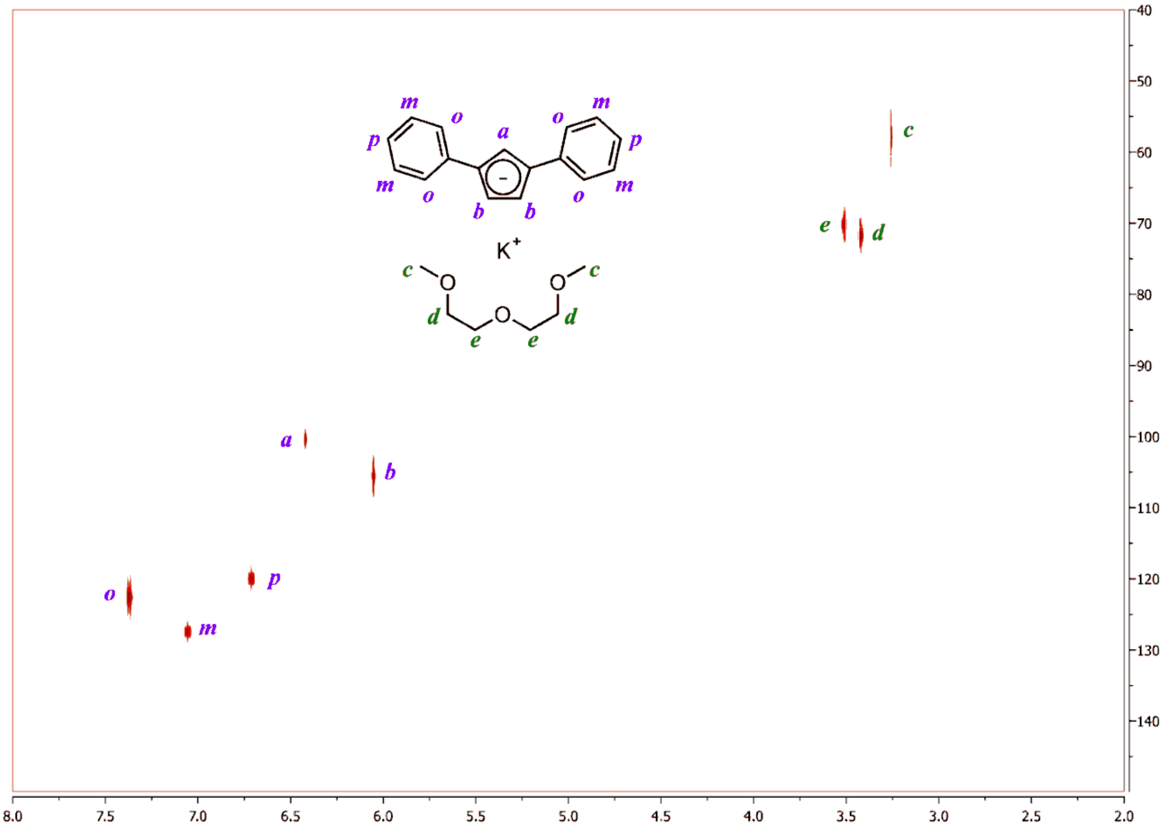

Figure S12. The ^{1}H-^{13}C GHSQC NMR spectrum of [K(diglyme)(1,3-$Ph_2C_5H_3$)], (**1a**), in THF_{d8} at 303K.

S2.2. [K(thf)(1,2,4-$Ph_3C_5H_2$)], (2).

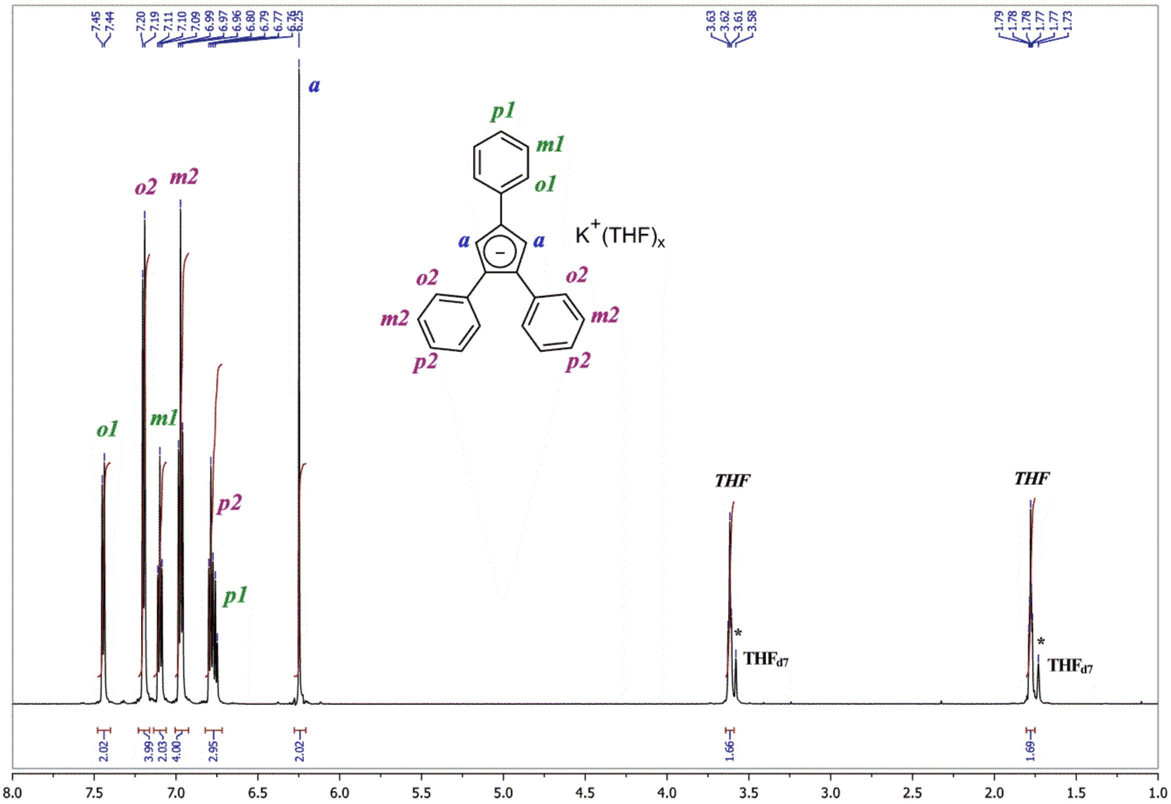

Figure S13. The ^{1}H NMR spectrum of [K(thf)$_x$(1,2,4-$Ph_3C_5H_2$)], (**2**), in THF_{d8} at 600MHz and 303K. Starred peaks are residual peaks from the deuturated solvent.

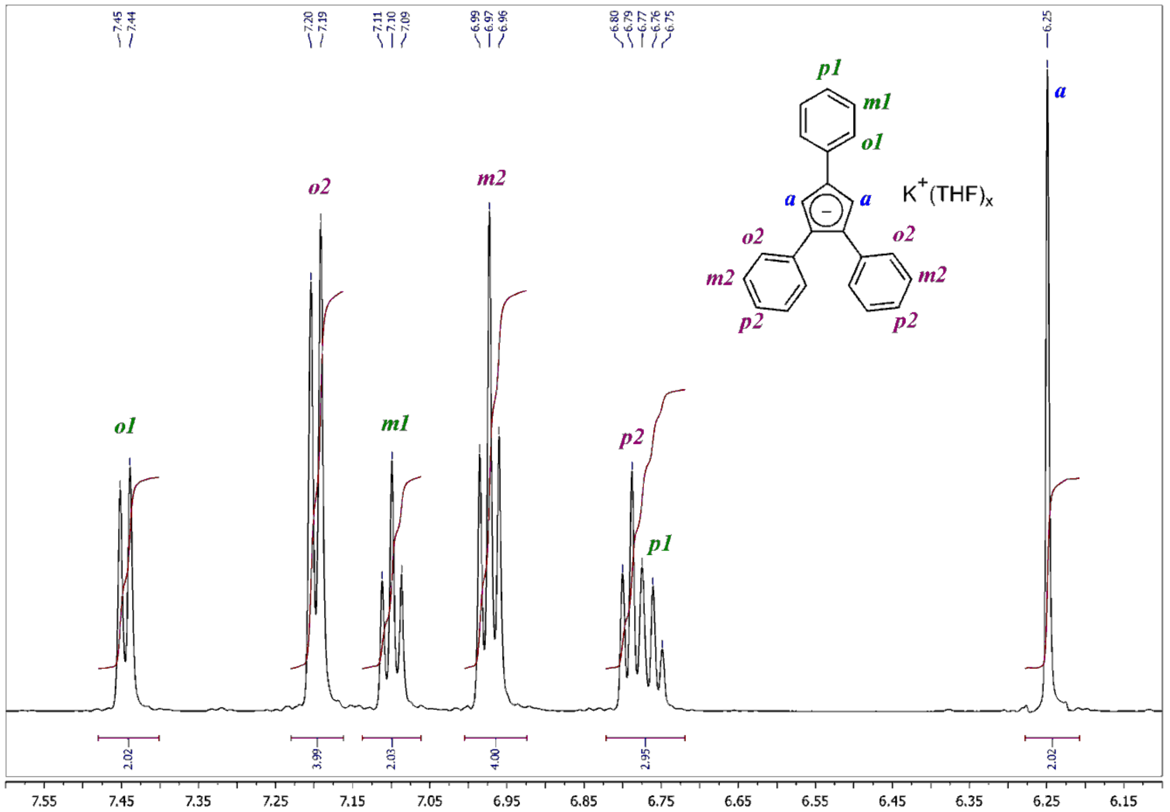

Figure S14. The enlarged aromatic region for the ^{1}H NMR spectrum of [K(thf)$_x$(1,2,4-$Ph_3C_5H_2$)], (**2**), in THF_{d8} at 600MHz.

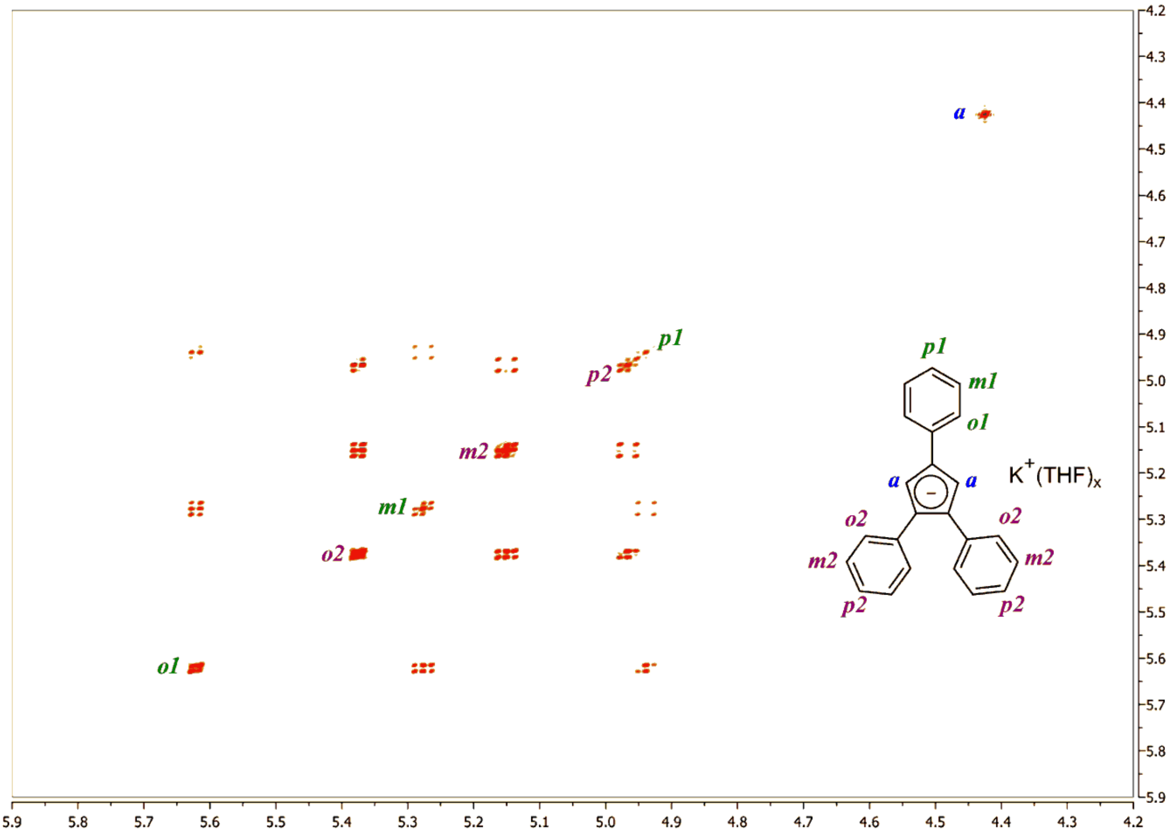

Figure S15. The aromatic region in the ^{1}H-^{1}H COSY NMR spectrum of [K(thf)$_x$(1,2,4-$Ph_3C_5H_2$)], (**2**), in THF$_{d8}$.

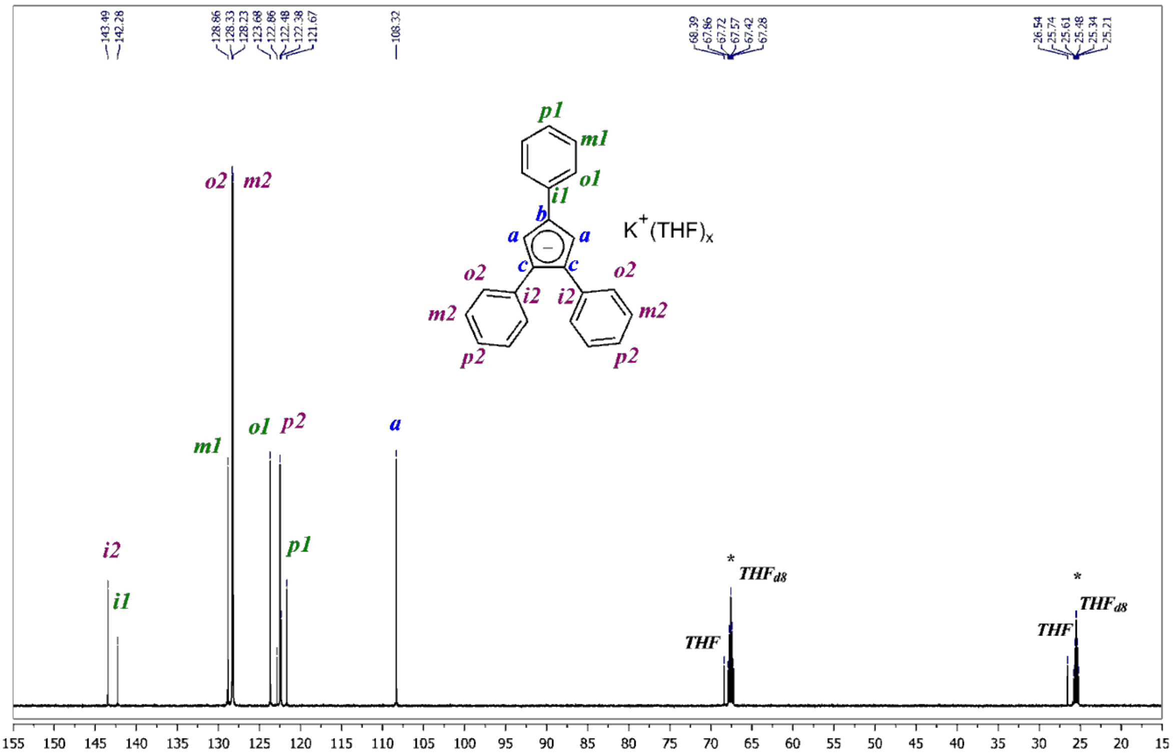

Figure S16. The $^{13}C\{^{1}H\}$ NMR spectrum of [K(thf)$_x$(1,2,4-$Ph_3C_5H_2$)], (**2**), in THF$_{d8}$ at 150MHz and 303K. Starred peaks are residual peaks from the deuturated solvent.

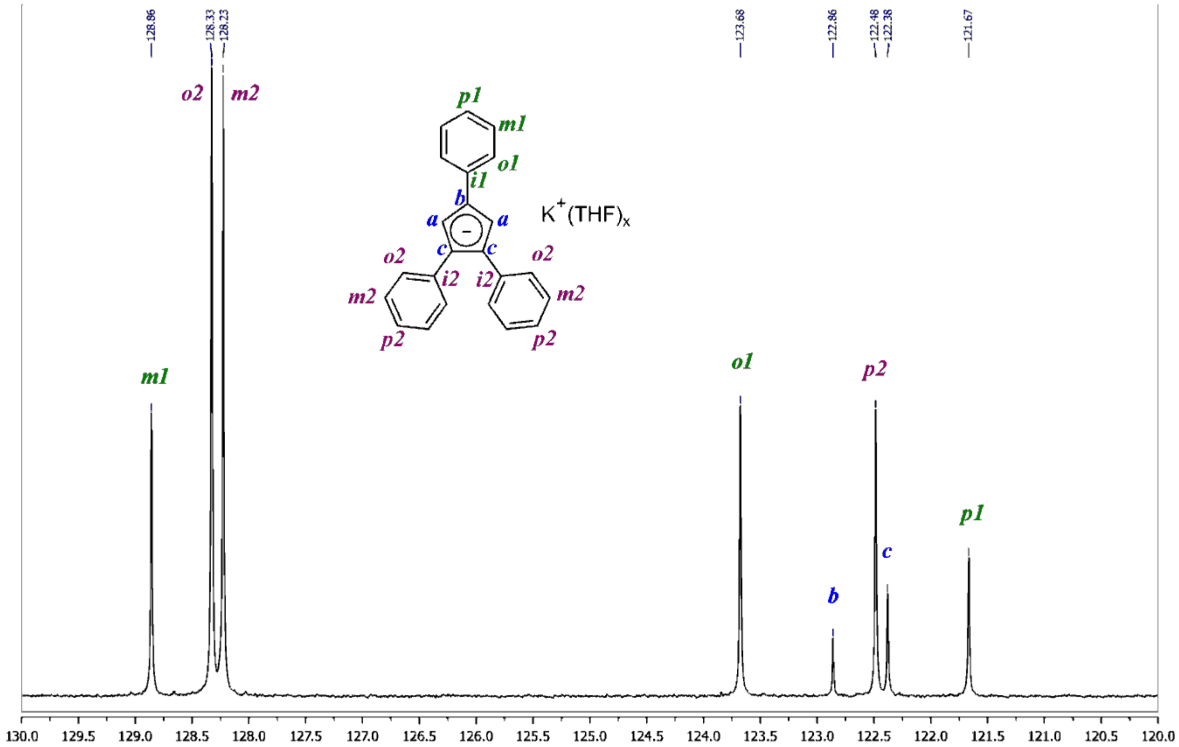

Figure S17. The enlarged region from 120 to 130 ppm for the $^{13}C\{^{1}H\}$ NMR spectrum of $[K(thf)_x(1,2,4\text{-}Ph_3C_5H_2)]$, (**2**), in THF_{d8} at 150MHz and 303K.

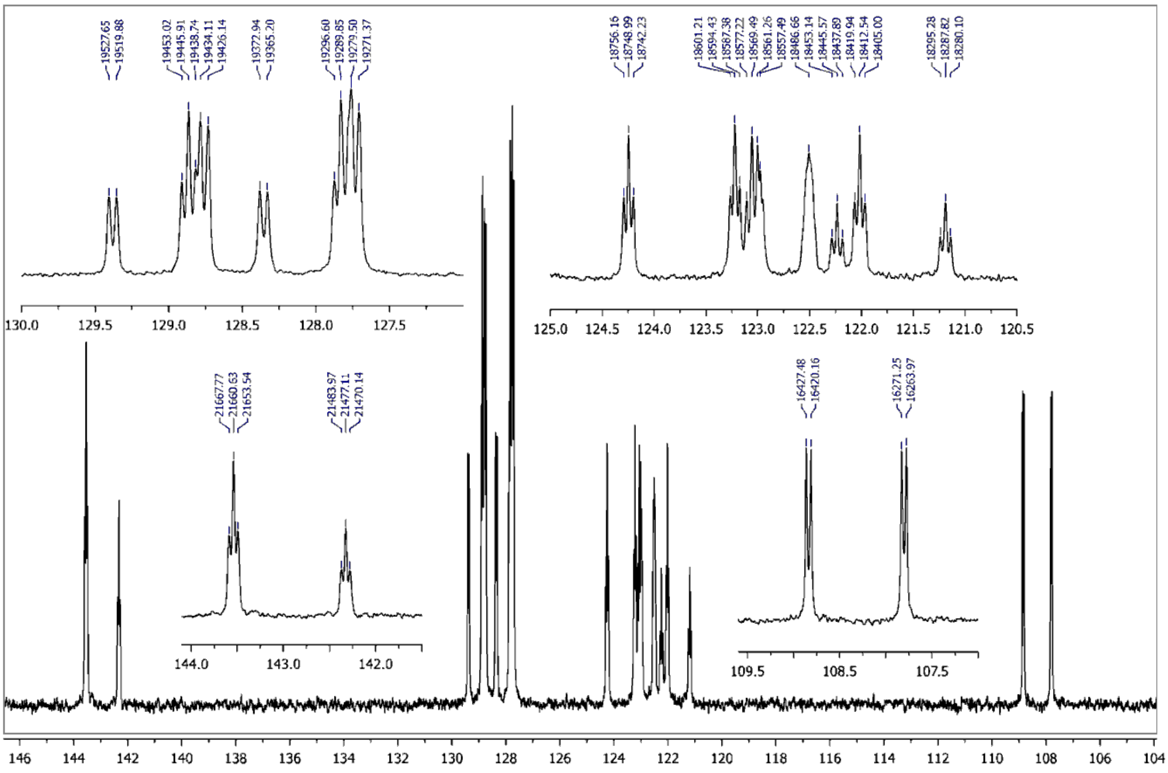

Figure S18. The ^{13}C NMR spectrum of $[K(thf)_x(1,2,4\text{-}Ph_3C_5H_2)]$, (**2**), in THF_{d8} at 151MHz and 303K.

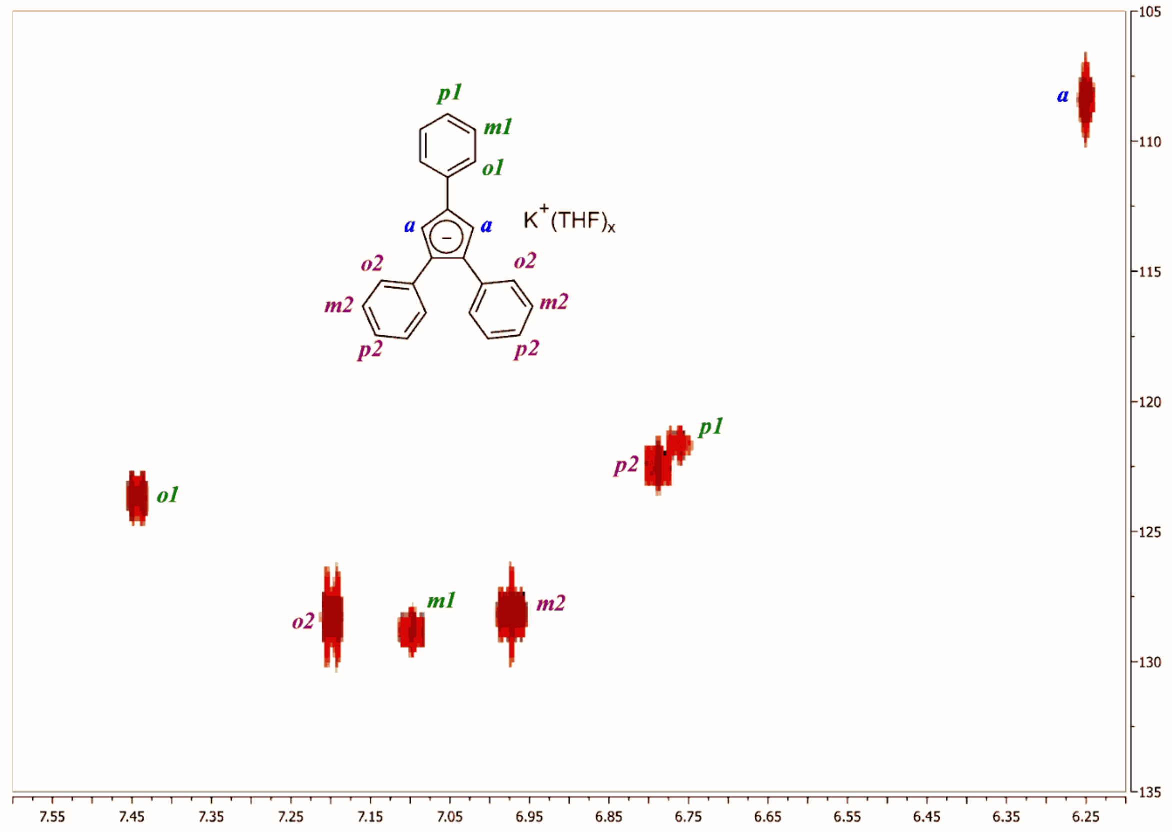

Figure S19. The ^{1}H-^{13}C GHSQC NMR spectrum of $[K(thf)_x(1,2,4\text{-}Ph_3C_5H_2)]$, (**2**), in THF_{d8}.

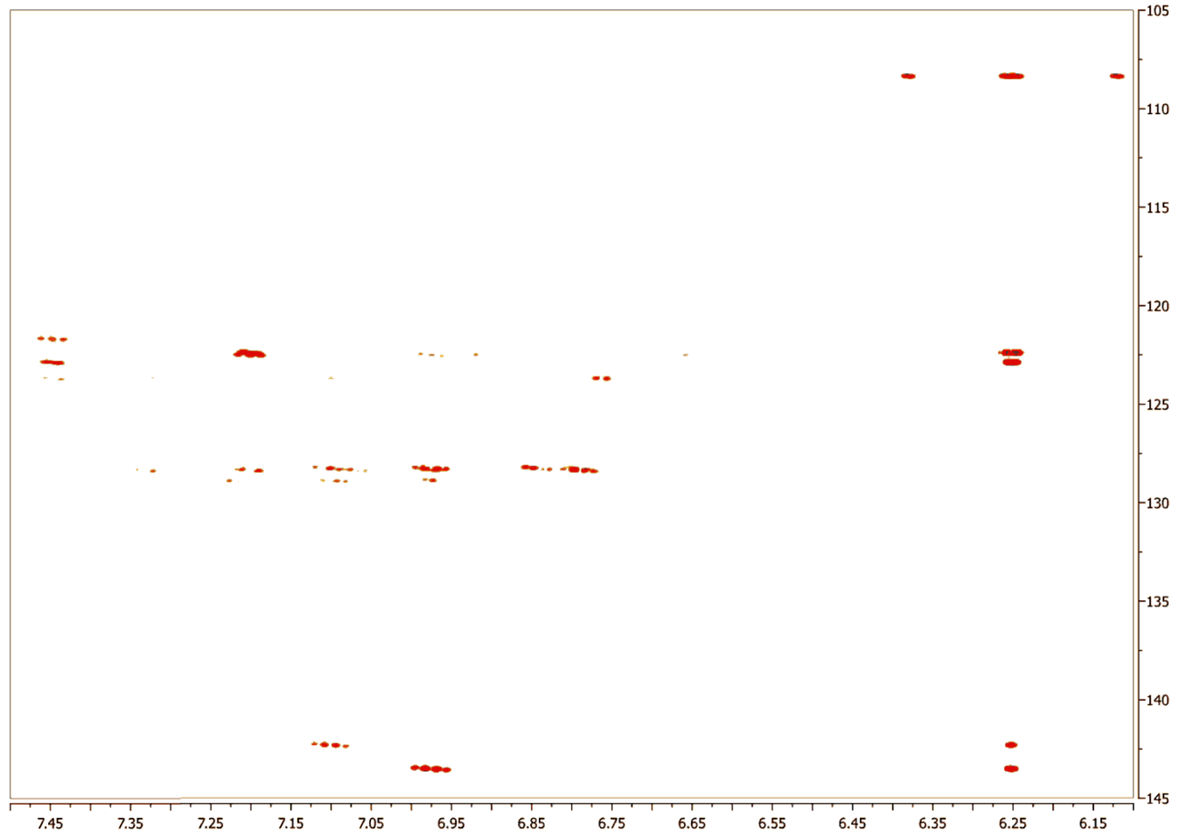

Figure S20. The ^{1}H-^{13}C HMBC NMR spectrum of $[K(thf)_x(1,2,4\text{-}Ph_3C_5H_2)]$, (**2**), in THF_{d8}.

S2.3. [K(thf)$_{0.4}$(1,2-Ph$_2$-4-(2-MeOC$_6$H$_4$)C$_5$H$_3$)], (3).

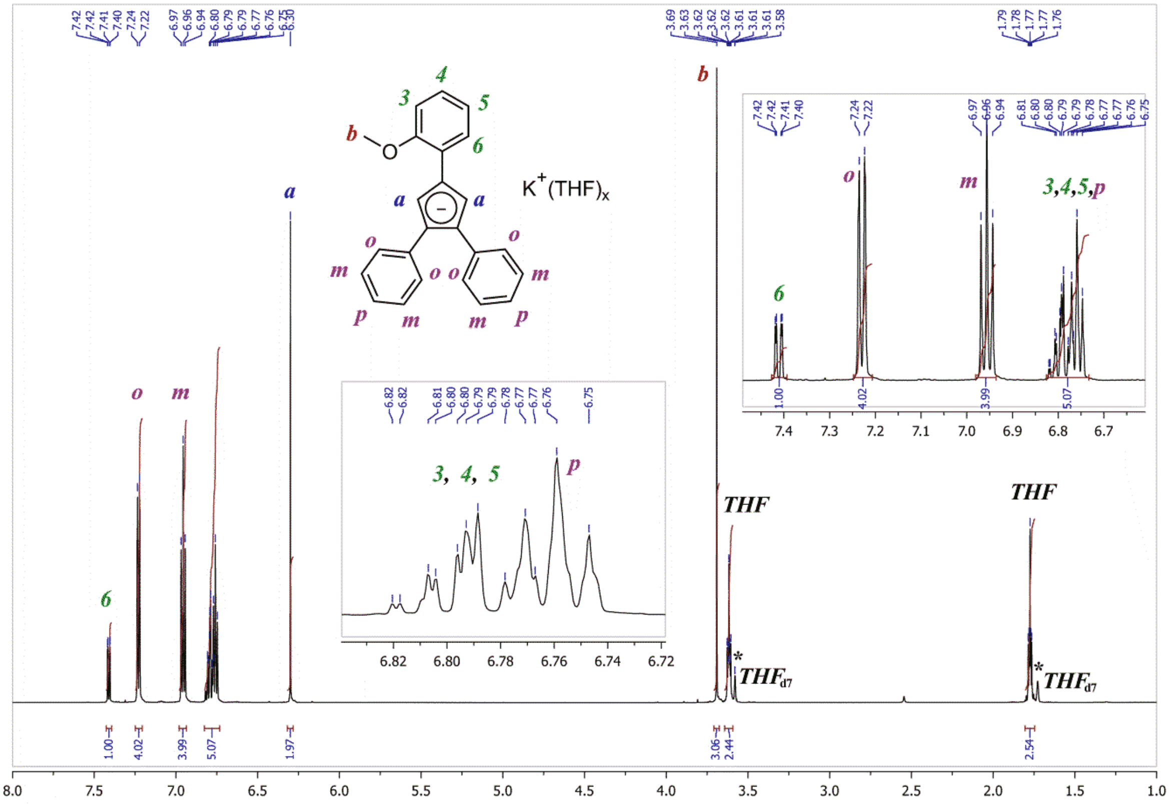

Figure S21. The ^{1}H NMR spectrum of [K(thf)$_x$(1,2-Ph$_2$-4-(2-MeOC$_6$H$_4$)C$_5$H$_3$)], (**3**), in THF$_{d8}$ at 600MHz and 303K. Starred peaks are residual peaks from the deuturated solvent.

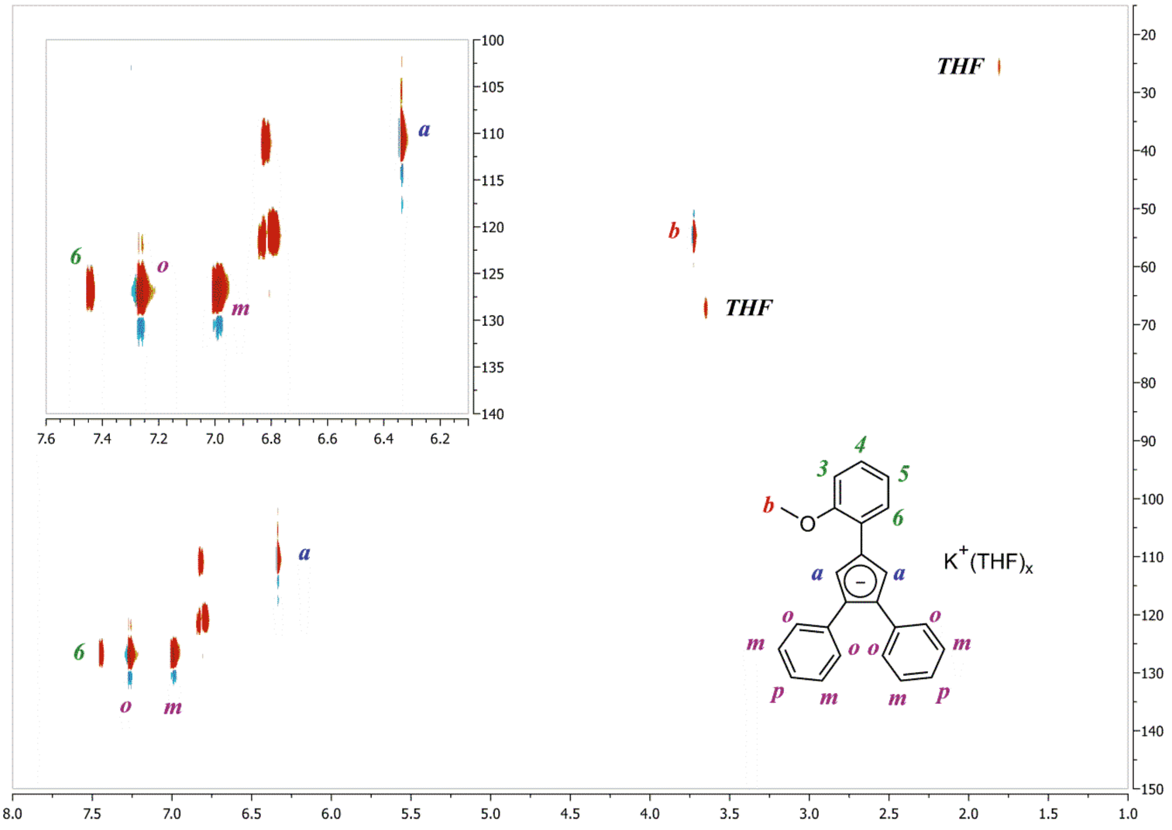

Figure S22. The ^{1}H-^{1}H COSY NMR spectrum of [K(thf)$_x$(1,2-Ph$_2$-4-(2-MeOC$_6$H$_4$)C$_5$H$_3$)], (**3**), in THF$_{d8}$.

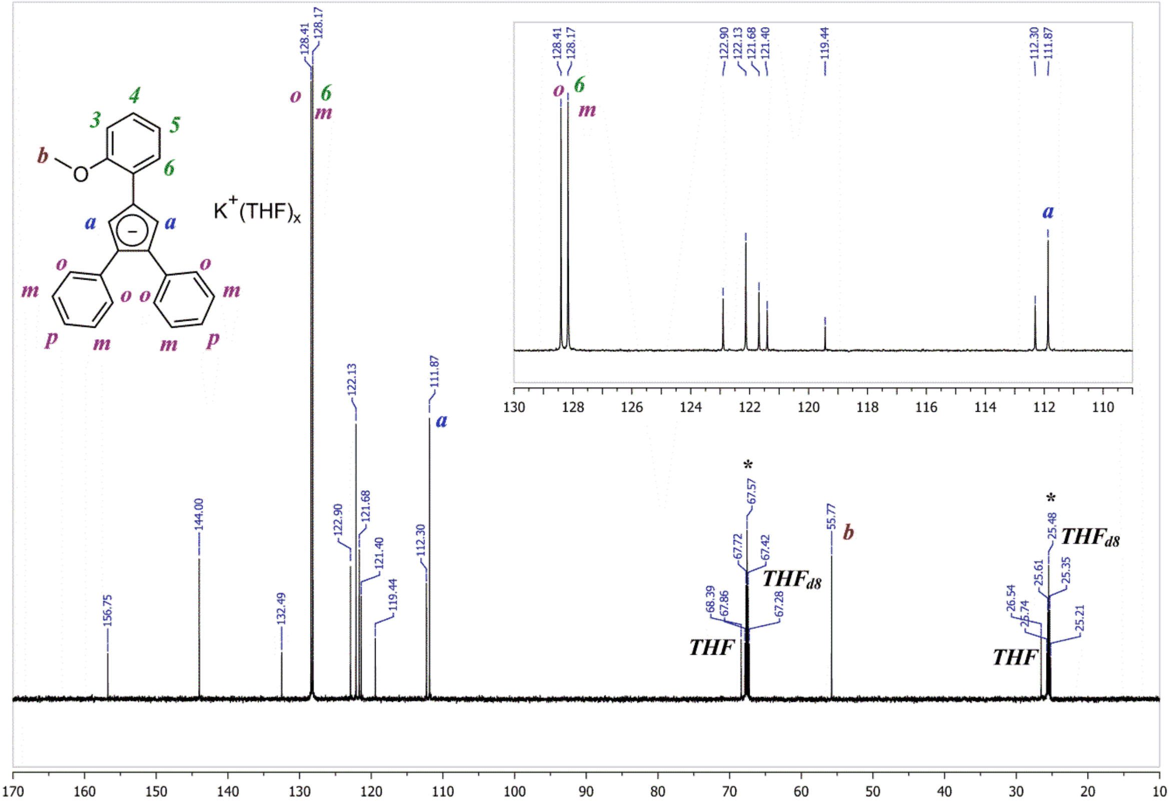

Figure S23. The $^{13}C\{^{1}H\}$ NMR spectrum of $[K(thf)_x(1,2\text{-}Ph_2\text{-}4\text{-}(2\text{-}MeOC_6H_4)C_5H_3)]$, (**3**), in THF_{d8} at 150MHz and 303K. Starred peaks are residual peaks from the deuturated solvent.

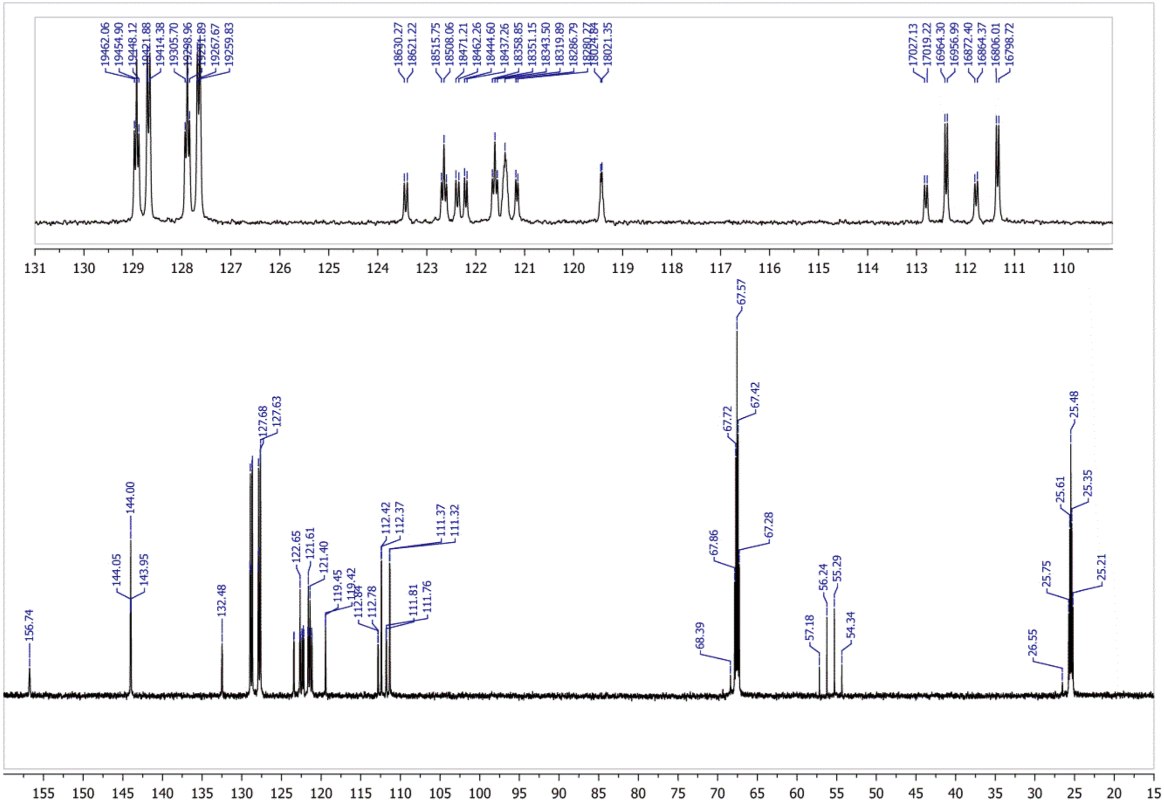

Figure S24. The ^{13}C NMR spectrum of $[K(thf)_x(1,2\text{-}Ph_2\text{-}4\text{-}(2\text{-}MeOC_6H_4)C_5H_3)]$, (**3**), in THF_{d8} at 150MHz and 303K. Starred peaks are residual peaks from the deuturated solvent.

S2.4. [K(thf)0.4(1,2-Ph2-4-(4-MeOC6H4)C5H3)], (4a).

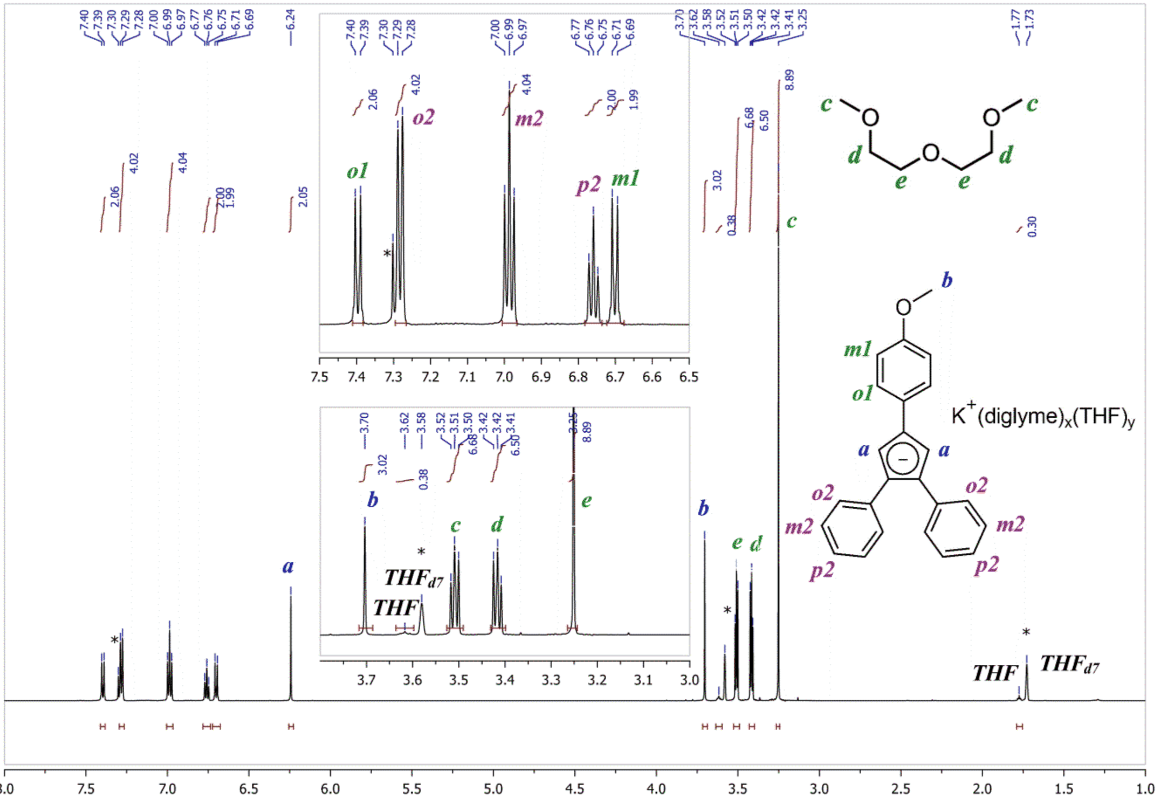

Figure S25. The ^{1}H NMR spectrum of [K(thf)x(1,2-Ph2-4-(4-MeOC6H4)C5H3)], (**4a**), in THFd8 at 600MHz and 303K. Starred peaks are residual peaks from the deuturated solvent.

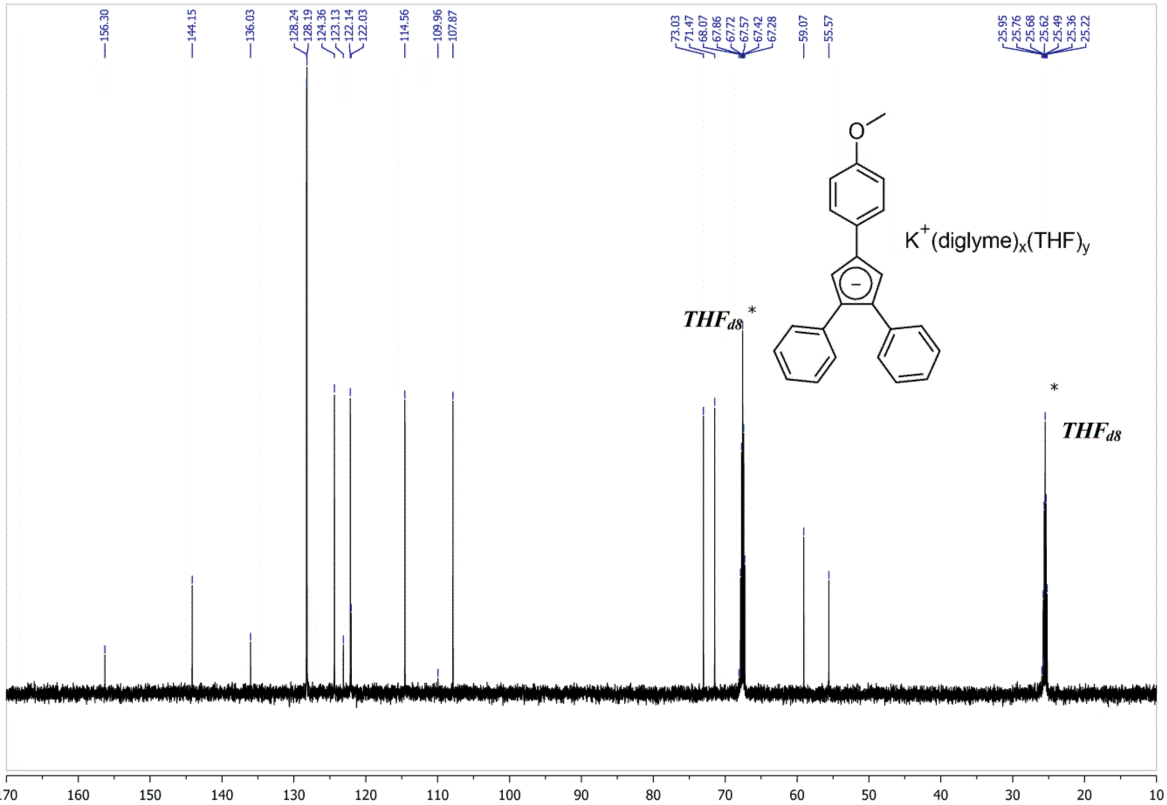

Figure S26. The $^{13}C\{^{1}H\}$ NMR spectrum of [K(thf)x(1,2-Ph2-4-(4-MeOC6H4)C5H3)], (**4**), in THFd8 at 150MHz and 303K. Starred peaks are residual peaks from the deuturated solvent.

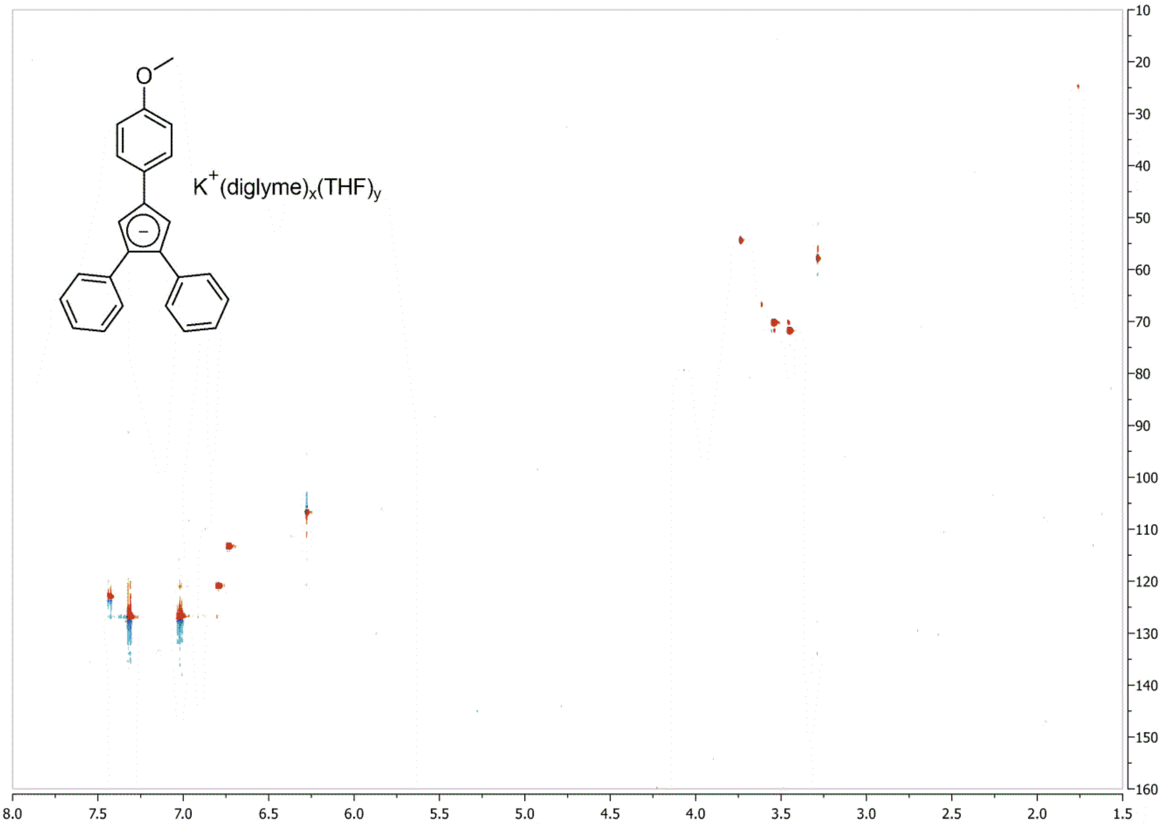

Figure S27. The ^{1}H-^{13}C GHSQC NMR spectrum of [K(thf)$_x$(1,2-Ph$_2$-4-(4-MeOC$_6$H$_4$)C$_5$H$_3$)], (**4a**), in THF$_{d8}$.

S3. Additional structural details on crystal structures of 2b, 4a and 2a.

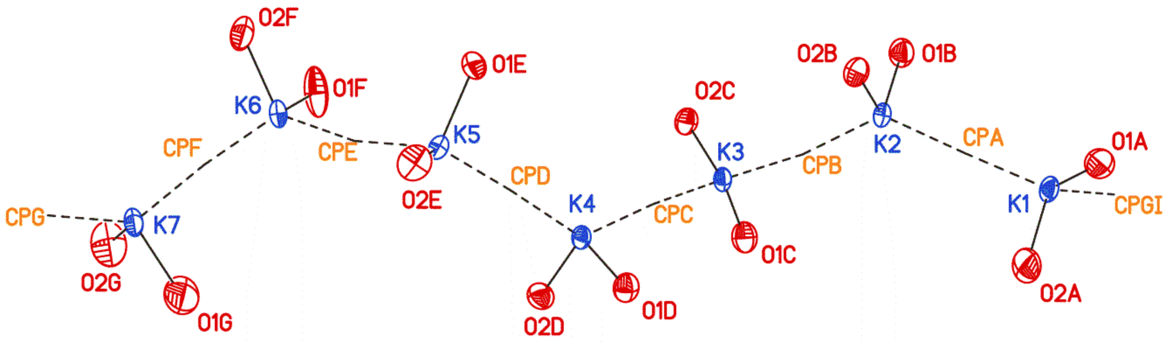

Figure S28. The chain structure of the asymmetric unit of **2b**. All H and C atoms are omitted. Cp-centroids are labeled as CPA through CPG. Displacement ellipsoids are drawn at a 30% probability level.

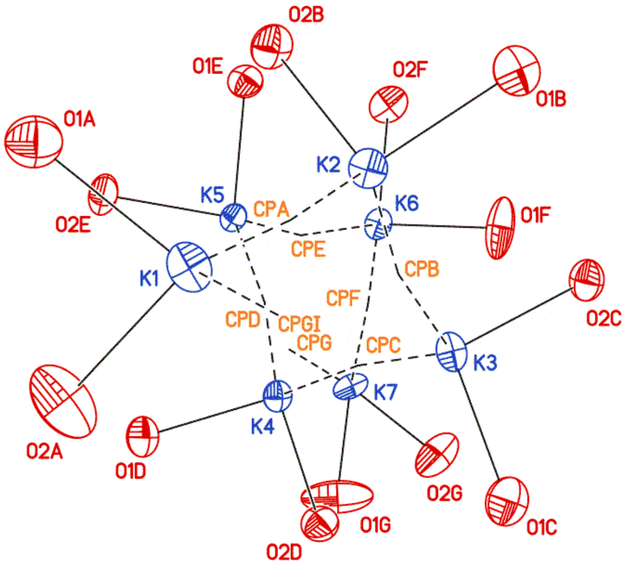

Figure S29. A view along an imaginary axis of the 1D chain of **2b** displays a helix-like structure. All H and C atoms are omitted; Cp-centroids are labeled as CPA through CPG. Displacement ellipsoids are drawn at a 30% probability level.

Table S1. Cp-Ph rotation angles (°) in **2b**

Cp (C1..C5)	Ph1 (C6..C11)	Ph2 (C12..C17)	Ph3 (C18..C23)
A	28.00(10)	41.73(13)	3.75(11)
B	31.44(15)	34.49(14)	3.5(2)
C	38.65(10)	32.89(18)	2.9(2)
D	40.71(17)	28.65(14)	0.9(2)
E	40.57(12)	24.31(17)	0.63(18)
F	38.34(16)	36.18(12)	3.91(12)
G	39.08(18)	34.86(18)	0.8(3)

Table S2. Cp-Ph rotation angles (°) in **4a**

Cp (C1..C5)	Ph1 (C6..C11)	Ph2 (C12..C17)	Ph3 (C18..C23)
A	39.2(2)	26.16(14)	3.87(11)
B	17.1(3)	43.2(2)	4.0(3)
C	24.3(3)	40.4(3)	6.2(3)
D	28.93(14)	40.3(2)	3.14(12)
E	25.9(4)	38.4(3)	9.1(4)
F	29.37(16)	37.20(19)	8.60(17)
G	35.2(2)	33.2(3)	1.9(4)

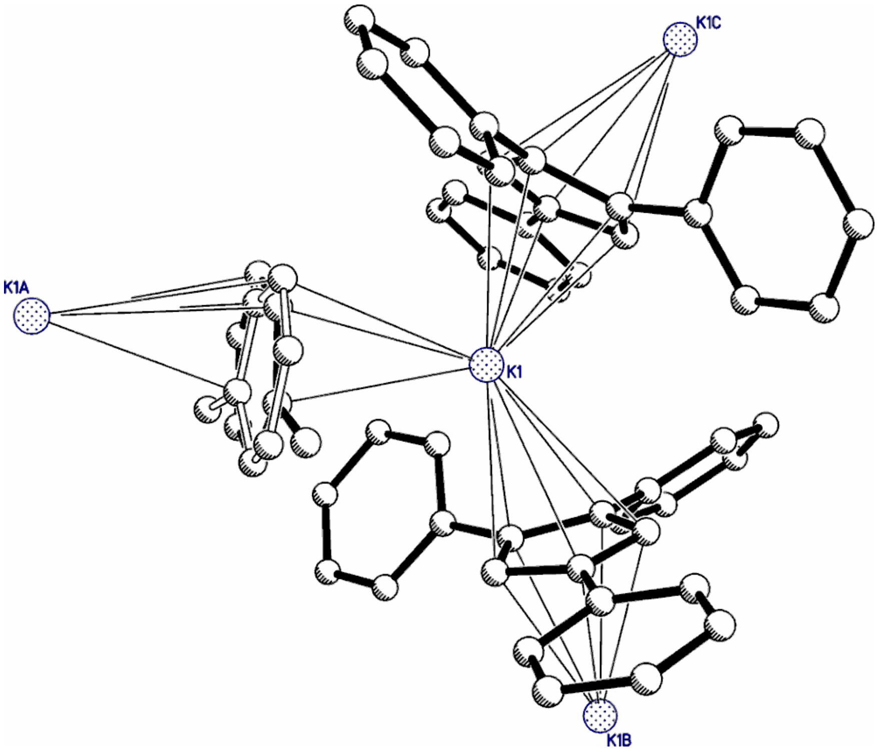

Figure S30. Ligand coordination modes in **2a**. A disorder of the coordinated toluene is shown. Atoms are drawn as spheres of fixed radii for clarity.

Table S3. Potassium coordination numbers, CN_K*

Compound	CN_K
**1a**	9
**2a**	8**
**2b**	8
**3**	8
**4a**	8 / 9***

* Potassium coordination numbers were formally calculated based on numbers of electron pair donated by ligands according to the literature: Elschenbroich, C., *Organometallics, 3rd, Completely Revised and Extended Edition*. 3rd ed.; Wiley-VCH**2016**; 817 pages.

** Two short K···C contacts with a Ph ring and two K···C contacts with toluene have been also accounted.

*** CN_K = 8 for moiety {K(κ^{2}O,O'-diglyme)[η^5-1,2-Ph_2-(4-$MeOC_6H_4$)C_5H_2]$_2$} (atom K6 and a major component of a disorder – atoms O2F, O3F) and for moiety {K(THF)$_2$[η^5-1,2-Ph_2-(4-$MeOC_6H_4$)C_5H_2]$_2$} (atom K7 and a major component of a disorder – atoms O5G, O6G). CN_K = 9 for other moieties {K(κ^{3}O,O',O"-diglyme)[η^5-1,2-Ph_2-(4-$MeOC_6H_4$)C_5H_2]$_2$}.
